# Genealogy of the neurodegenerative diseases based on a meta-analysis of age-stratified incidence data

**DOI:** 10.1038/s41598-020-75014-8

**Published:** 2020-11-03

**Authors:** Daniela Gerovska, Haritz Irizar, David Otaegi, Isidre Ferrer, Adolfo López de Munain, Marcos J. Araúzo-Bravo

**Affiliations:** 1 Computational Biology and Systems Biomedicine Group, Biodonostia Health Research Institute, Calle Doctor Beguiristain S/N, 20014 San Sebastián, Spain; 2 Computational Biomedicine Data Analysis Platform, Biodonostia Health Research Institute, Calle Doctor Beguiristain S/N, 20014 San Sebastián, Spain; 3 Icahn Institute for Genomics & Multiscale Biology and Department of Genetics and Genomic Sciences, Icahn School of Medicine at Mount Sinai, New York, NY 10029 USA; 4 Division of Psychiatry, Faculty of Brain Sciences, University College London, London, WC1E 6BT UK; 5 Instituto Biodonostia-Hospital Universitario Donostia, San Sebastián, Gipuzkoa Spain; 6 Departamento de Patología y Terapéutica Experimental, Universidad de Barcelona, CIBERNED, Hospitalet de LLobregat, Barcelona, Spain; 7 IKERBASQUE, Basque Foundation for Science, Calle María Díaz Harokoa 3, 48013 Bilbao, Spain; 8 CIBER of Frailty and Healthy Aging (CIBERfes), Madrid, Spain; 9 Computational Biology and Bioinformatics Group, Max Planck Institute for Molecular Biomedicine, Röntgenstr. 20, 48149 Münster, Germany

**Keywords:** Computational biology and bioinformatics, Neuroscience, Systems biology, Diseases, Medical research, Pathogenesis, Risk factors

## Abstract

While the central common feature of the neurodegenerative diseases (NDs) is the accumulation of misfolded proteins, they share other pathogenic mechanisms. However, we miss an explanation for the onset of the NDs. The mechanisms through which genetic mutations, present from conception are expressed only after several decades of life are unknown. We aim to find clues on the complexity of the disease onset trigger of the different NDs expressed in the number of steps of factors related to a disease. We collected brain autopsies on diseased patients with NDs, and found a dynamic increase of the ND multimorbidity with the advance of age. Together with the observation that the NDs accumulate multiple misfolded proteins, and the same misfolded proteins are involved in more than one ND, motivated us to propose a model for a genealogical tree of the NDs. To collect the dynamic data needed to build the tree, we used a Big-data approach that searched automatically epidemiological datasets for age-stratified incidence of NDs. Based on meta-analysis of over 400 datasets, we developed an algorithm that checks whether a ND follows a multistep model, finds the number of steps necessary for the onset of each ND, finds the number of common steps with other NDs and the number of specific steps of each ND, and builds with these findings a parsimony tree of the genealogy of the NDs. The tree discloses three types of NDs: the stem NDs with less than 3 steps; the trunk NDs with 5 to 6 steps; and the crown NDs with more than 7 steps. The tree provides a comprehensive understanding of the relationship across the different NDs, as well as a mathematical framework for dynamic adjustment of the genealogical tree of the NDs with the appearance of new epidemiological studies and the addition of new NDs to the model, thus setting the basis for the search for the identity and order of these steps. Understanding the complexity, or number of steps, of factors related to disease onset trigger is important prior deciding to study single factors for a multiple steps disease.

## Introduction

Neurodegeneration is the set of complex biological processes that over a long period of time lead to neuronal or glia, pericytes, or both, malfunction and cellular death. While the central common feature of the Neurodegenerative Diseases (NDs) is the accumulation of misfolded proteins^1,2^, they share other pathogenic mechanisms like mitochondrial dysfunction, oxidative stress, defective protein quality-control and degradation pathways, stress granules and maladaptive immune responses^2,3^. The genoprotein hypothesis suggests that every ND is produced by the preferential deposit with characteristic progression patterns of a particular protein in vulnerable places of the nervous system. A large body of evidence^1^ shows that the NDs share features at molecular and histological level, with protein aberrant aggregation resulting neuronal death among all, and concluded that the NDs might have common mechanisms^4^. Several proteins are involved in two or more NDs^5–31^. A general protein distribution mechanism explains how protein damage accumulated with age is asymmetrically distributed during Neural Stem Cells (NSCs) division in rodents, where dividing NSCs establish a diffusion barrier in the endoplasmic reticulum membrane that restricts damaged proteins to one daughter cell, leaving the other with intact molecules. But with age this diffusion barrier weakens in response to impairment of lamin-associated nuclear envelope constituents, so that replicating NSCs of older animals are less able to exclude damaged proteins than of the younger ones^32^.

The adult onset of NDs is still unexplained. The mechanisms through which genetic mutations present from conception are expressed only after several decades of life are unknown. Cancer researchers have tried to explain why cancer would reveal itself mostly in adult age and would show an increasing incidence with age with a multistep pathogenic model of cancer where several subsequent mutations or pathogenic events, are necessary to trigger the disease, and have modeled the patterns of cancer incidence with age to infer the average number of pathogenic steps required^33,34^. The multistep approach was further applied to Amyotrophic Lateral Sclerosis (ALS)^35^ and Alzheimer's disease (AD)^36^. However, an integrative method explaining the relationship among all NDs was missing.

**Figure 1:**
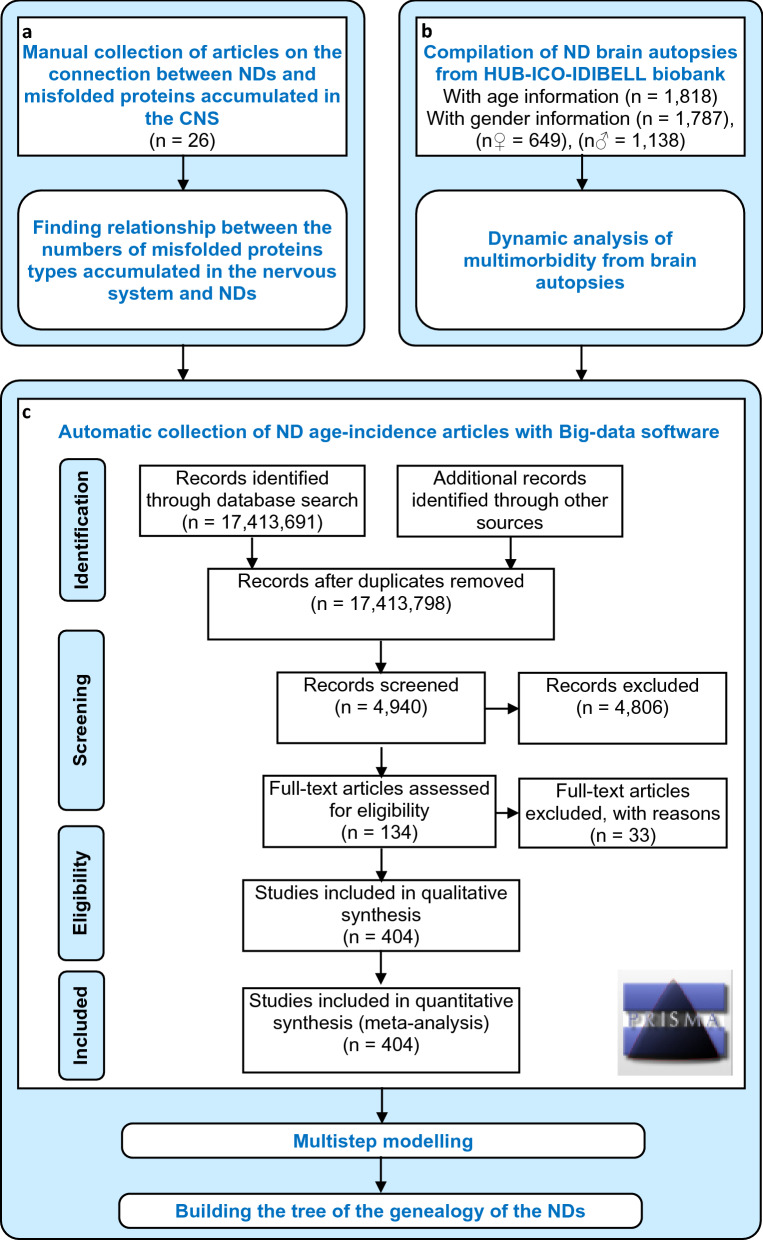
Experimental design. (**a**) Manual collection of articles to analyze the relationship between the numbers of different types of misfolded proteins accumulated in the nervous system and neurodegenerative diseases (NDs). (**b**) Brain autopsies collection from HUB-ICO-IDIBELL biobank, Spain, to analyze the dynamics of the brain multimorbidity with the advance of age. (**c**) Automatic search with our Big-data software of age-stratified incidence data articles and manual curation and data extraction for building of the NDs tree. The flow diagram follows the Preferred Reporting Items for Systematic Reviews and Meta-Analyses (PRISMA)-style guidelines^51^.

To develop such method, firstly, from manual collection of articles, we analyzed the relationship between the numbers of different types of misfolded proteins accumulated in the Central Nervous System and NDs to confirm that the NDs shared multiple misfolded proteins and that the same misfolded proteins are involved in different NDs (Fig. 1a). Secondly, we collected brain autopsies from the HUB-ICO-IDIBELL biobank, Spain, to analyze the dynamics of the brain multimorbidity with the advance of age (Fig. 1b). Most importantly, we performed an automatic search with our Big-data software of age-stratified incidence data articles, then a manual curation and data extraction for building of the tree of NDs (Fig. 1c).

We collected data on age-stratified incidence of the major NDs: AD, Parkinson's disease (PD), Huntington's disease (HD), ALS, Fronto Temporal Dementia (FTD), as well as Dementia with Lewy Bodies (DLB), Parkinsonism (PDM), Parkinson’s Disease with Dementia (PDD) and Creutzfeldt–Jakob disease (CJD), and under the assumption that they share pathogenic mechanisms we studied whether such mechanisms have left a fingerprint on the dynamics of their incidence patterns with age and whether such fingerprints can provide insights about the ND triggering mechanisms. We used as a control Multiple Sclerosis (MS), a disease with a neurodegenerative component, though not as central as in the diseases mentioned above.

## Results

### Several misfolded proteins are involved in two or more NDs

**Figure 2:**
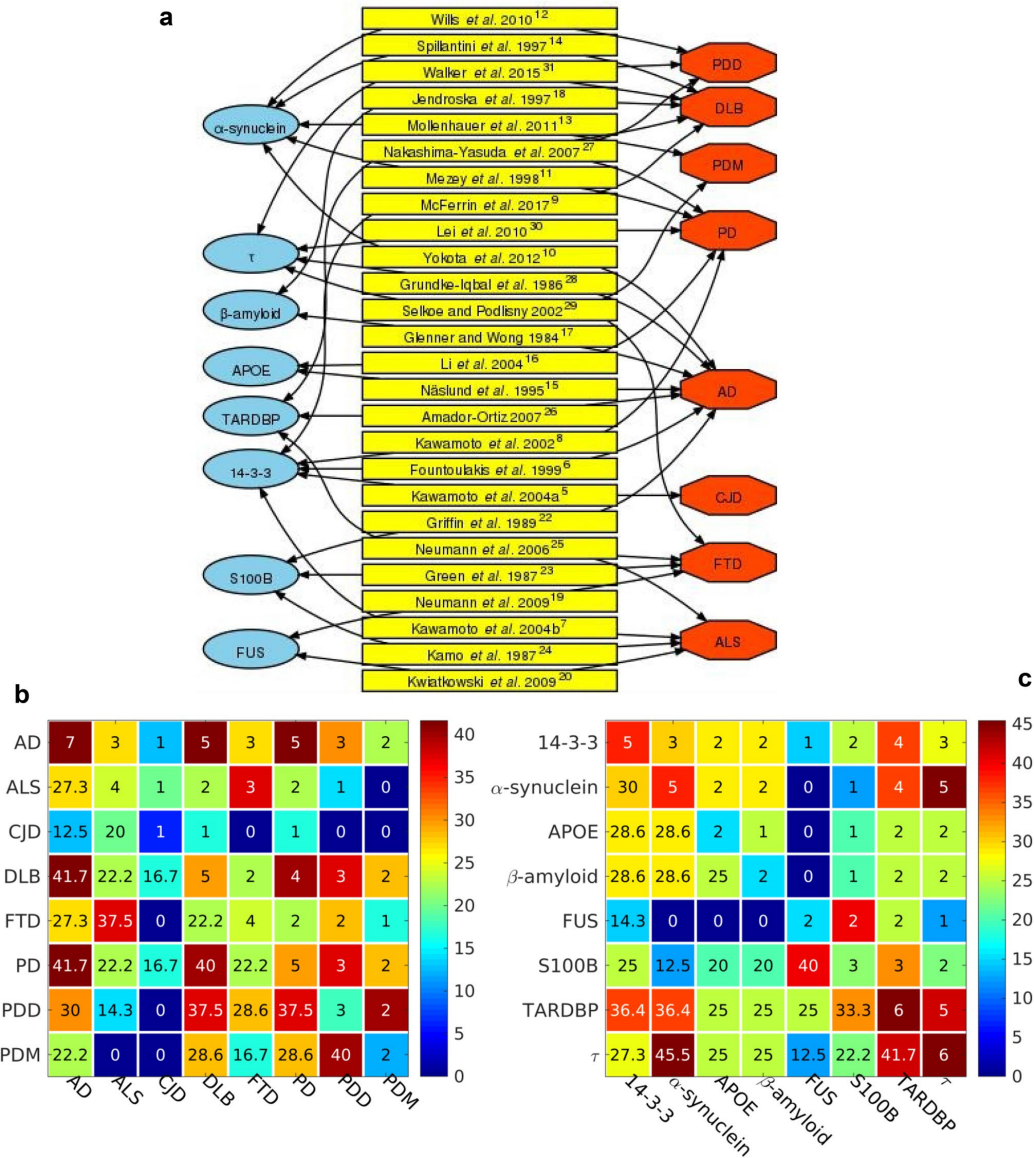
Relationship between the numbers of different types of misfolded proteins accumulated in the nervous system and neurodegenerative diseases (NDs). (**a**) Protein—ND network. Blue ellipses, green rectangles and red octahedra represent proteins, references and NDs, respectively. The arrows mark relations reported in the literature. (**b**) Heatmap of the number of protein types (upper triangular elements) and the percentage of protein types (lower triangular elements) accumulated in two NDs. The diagonal shows the number of different proteins reported to have affected each ND. The color bar to the right gives a color codification of the percentage of protein types. Bluer and redder colors correspond to lower and higher percentages of protein types, respectively. (**c**) Heatmap of the number of NDs (upper triangular elements) and the percentage of NDs (lower triangular elements) associated with two protein types. The diagonal shows the number of NDs reported to have been affected by each protein type. The color bar to the right gives a color codification of the percentage of NDs. Bluer and redder colors correspond to lower and higher percentages of NDs, respectively. NDs: Amyotrophic Lateral Sclerosis (ALS), Alzheimer's disease (AD), Parkinson's disease (PD), Fronto Temporal Dementia (FTD), Dementia with Lewy Bodies (DLB), Parkinsonism (PDM), Parkinson’s Disease with Dementia (PDD), Creutzfeldt–Jakob disease (CJD).

We studied the relationship between the numbers of different types of misfolded proteins accumulated in the nervous system and neurodegenerative diseases (NDs) through article collection (Fig. 1a), and built a protein—ND network (Fig. 2a) of proteins, references and NDs with arrows marking the relations reported in the literature. 14-3-3 proteins are implicated in CJD^5^, AD^6^, ALS^7^, PD^8^ and DLB^9^; α-synuclein is involved in AD^10^; and in synucleinopathies such as PD^11^, PDD^12^, PDM^13^ and DLB^14^; APOE is involved in AD^15^ and PD^16^; β-amyloid is involved in AD^17^ and DLB^18^; FUS is involved in Fronto Temporal Dementia (FTD)^19^ and ALS^20,21^ S100B is involved in AD^22^, FTD^23^ and ALS^24^; TARDBP/TDP-43 is involved in FTD, ALS^25^, AD^26^, DLB, PD and PDD^27^; τ is involved in tauopathies such as AD^28^, FTD and PDM^29^; in PD^30^, PDD and DLB^31^. The heatmap synthesis of the reference data on the protein types in NDs has a diagonal with the number of misfolded proteins associated with a certain ND (Fig. 2b). E.g. we found references on 7 misfolded proteins associated with AD, 5 with DLB and PD, 4 with ALS, FTD, 3 with PDD, 2 with PDM, and 1 with CJD. The upper triangular elements of the heatmap show the number of common misfolded proteins for a pair of NDs. E.g. the first upper diagonal row shows that AD and ALS, AD and FTD, AD and PDD, are associated with 3 common misfolded proteins, the same number but not necessarily the same 3 proteins. AD and DLB, and AD and PD are associated with 5 common proteins, while AD and PDM have 2 common proteins, and AD and CJD with only 1. The lower triangular elements of the heatmap represent the same data in percent, e.g. the AD and PD share 41.7% of their associated misfolded proteins. Another heatmap on the NDs associated with a misfolded protein (Fig. 2c) shows in its diagonal the number of NDs reported as affected by a certain misfolded protein type. E.g. TARDBP and τ are found to be accumulated in 6 NDs, 14–3-3 and α-synuclein in 5 NDs, S100B in 3 NDs, and APOE, β-amyloid, and FUS in 2 NDs. The upper diagonal shows the number of NDs in which 2 misfolded proteins are reported to be accumulated simultaneouly. E.g. TARDBP and τ are reported to accumulate in 5 NDs simultaneously. Another example of simultaneous accumulation of 2 misfolded proteins in 5 NDs, is of α-synuclein and τ. Although not exhaustive, the association data based on references shows that one ND is often associated with the accumulation of more than one misfolded proteins, and that two misfolded proteins are often associated with more than one NDs, all that suggesting that different NDs could share common mechanisms of onset.

### PCA analysis reveals three ND groups of age-stratified incidence profiles

**Figure 3:**
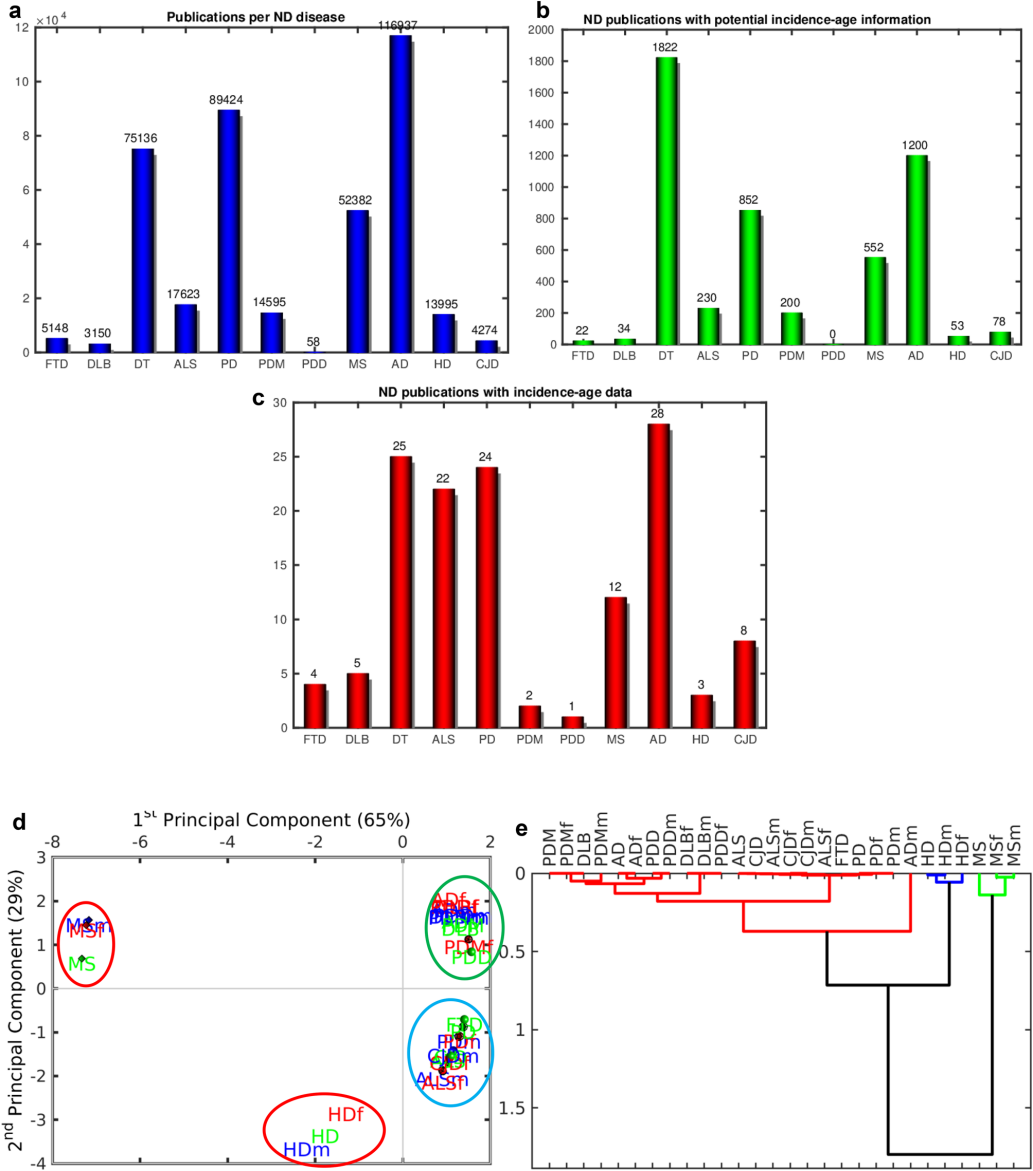
Compilation of age-stratified incidence data on neurodegenerative diseases (NDs). (**a**) Publications per ND in PubMed. (**b**) ND publications with potential age-stratified incidence information in PubMed. (**c**) ND publications processed with age-stratified incidence information. Non-parametric analysis of neurodegenerative disease age-stratified incidence profiles. (**d**) Principal Component Analysis (PCA). The female (f), male (m) and total samples are in red, blue and green, respectively. The ellipses mark clusters of NDs. (**e**) Hierarchical clustering of samples using the Pearson correlation metric and the average linkage method. NDs: Amyotrophic Lateral Sclerosis (ALS), Alzheimer's disease (AD), Parkinson's disease (PD), Huntington's disease (HD), Fronto Temporal Dementia (FTD), Dementia with Lewy Bodies (DLB), Parkinsonism (PDM), Parkinson’s Disease with Dementia (PDD), Creutzfeldt–Jakob disease (CJD), Multiple Sclerosis (MS).

To collect age-stratified incidence profiles we developed a software that scanned all the abstracts of PubMed which as of 31/1/2019 had registered 17,413,691 publications (Fig. 1c), and selected the ones with the name of one of the NDs in the title or abstract (Fig. 3a). Next, it filtered the ones with the words “incidence” and “age” in the title or abstract (Fig. 3b). Finally, we selected manually the publications with age-stratified incidence data on the NDs (Fig. 3c). The full list of references to the studies from which data on age-stratified incidence was extracted for each ND (Supplementary Table S1-S4), and meta-analysis forest plots (Supplementary Fig. S2-S29) are provided in the Supplementary Material.

We compared the patterns of age-stratified incidence profiles using hypothesis-free techniques such as principal component analysis (PCA) and hierarchical clustering. The typical NDs (AD, PD, ALS, FTD, PDM, PDD and CJD) have similar incidence patterns and group together in both PCA (Fig. 3d) and hierarchical clustering (Fig. 3e). All of them, except HD, have only positive values of the first principal component (PC) in the PCA explaining 65% of the variability of the disease age-stratified incidence profiles. Although the MS and HD groups have negative values of the first PC, they group away from each other, with contrasting positive and negative values, respectively, of the second PC of the PCA explaining 29% of the variability of the profiles. MS, however, has different dynamics in terms of the age-stratified incidence profiles, reflected by a well separated group in both the hierarchical clustering and the PCA, while HD forms a group close to the typical NDs and still away from them (Fig. 3d). In short, the typical NDs manifest similar dynamics of the age-stratified incidence profiles, while these dynamics in MS and to a certain degree in HD, follow a separate trend.

### Brain autopsies on diseased patients with NDs demonstrate a dynamic increase of the ND multimorbidity with the advance of age

**Figure 4:**
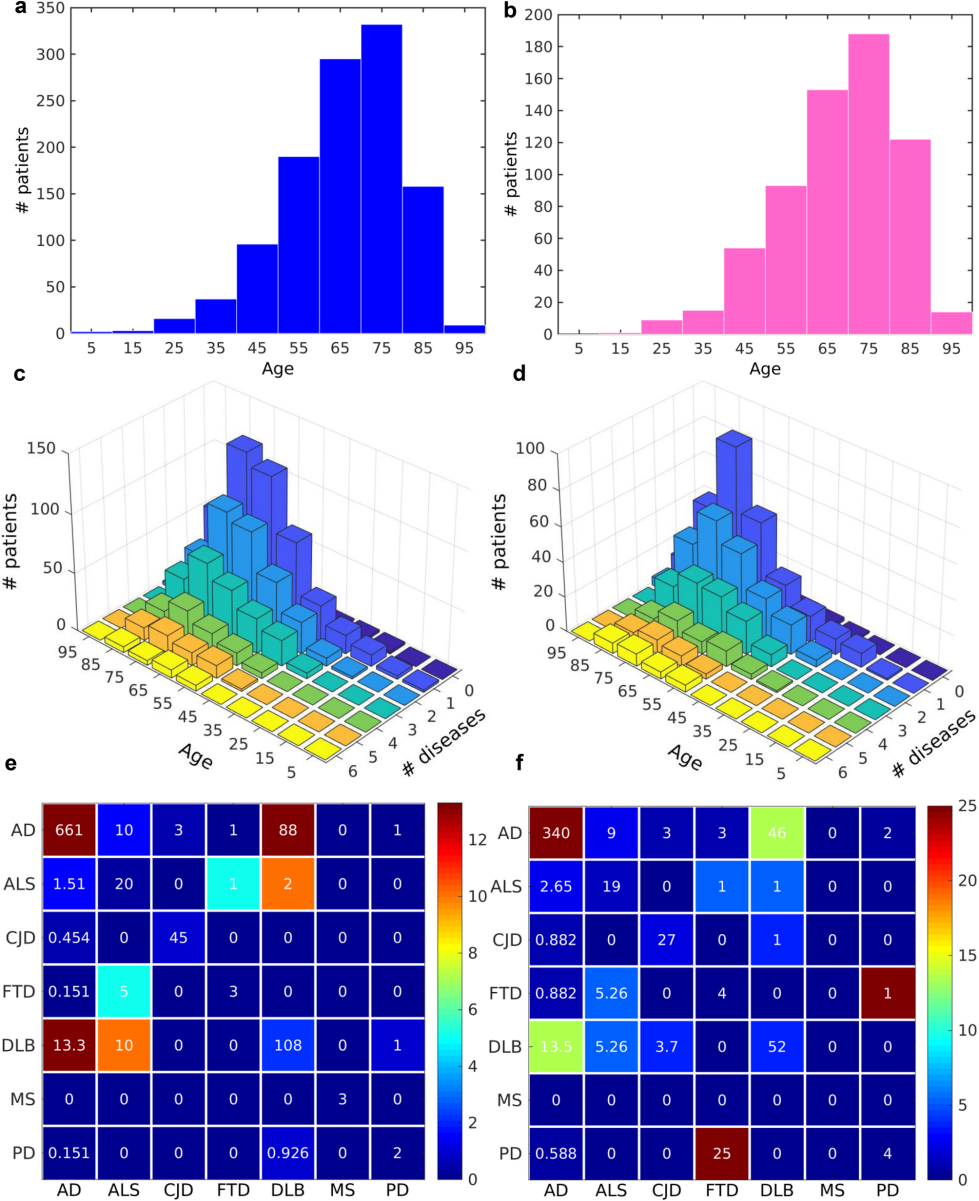
Dynamic analysis of multimorbidity from brain autopsies. Histogram of the distribution of the number of (**a**) male and (**b**) female patients per age. Number of (**c**) male and (**d**) female patients with a number of NDs for each age range in (**c**) male and (**d**) female. Number of: (**e**) male and (**e**) female patients (upper triangular elements) and percentage of patients (lower triangular elements) with at least two NDs. The diagonals show the number of patients with each disease. The color bar to the right gives a color codification of percentage of patients. Bluer and redder colors correspond to lower and higher percentages of patients, respectively. NDs: Amyotrophic Lateral Sclerosis (ALS), Alzheimer's disease (AD), Parkinson's disease (PD), Fronto Temporal Dementia (FTD), Dementia with Lewy Bodies (DLB), Creutzfeldt–Jakob disease (CJD), Multiple Sclerosis (MS).

In our study of brain autopsies on diseased patients with NDs, we collected a higher number of male than female cases. However, for both sexes the distributions of patients per age are similar (Fig. 4a,b). The maximum number of cases peaks in the age range [70, 80) and after that age the number drops due to the lack of samples (Fig. 4a,b). Our study demonstrates that the total number of detected NDs increases with the advance of age for both male and female (Fig. 4c,d). From the total of 649 autopsies corresponding to females, for those in the age range [70, 80), 56 (8.63%) have 2 NDs, 27 (4.16%) have 3, 12 (1.85%) have 4, 8 (1.23%) have 5, and 7 (1.08%) have even 6 NDs. From the total of 1,138 autopsies corresponding to males, for those in the age range [70, 80), 91 (8.00%) have 2 NDs, 57 (5.01%) have 3, 26 (2.28%) have 4, 15 (1.32%) have 5, and 4 (0.25%) have even 6 NDs. We searched for the most common ND comorbidities, independently of the age distribution, and we found a large number of diseased with several NDs, 13% males and 14% females had AD and DLB, 10% males and 5% females had ALS and DLB, 2% males and 3% females had ALS and AD, etc. (Fig. 4e,f). Additionally, numerous studies reported mixed brain pathologies in dementia^37,38^. This increase of the NDs comorbidity with the age could indicate the existence of common dynamic mechanism that triggers the onset of the NDs across the life span.

### Most of the datasets of age-stratified incidence profiles of NDs fit the multistep model

**Figure 5:**
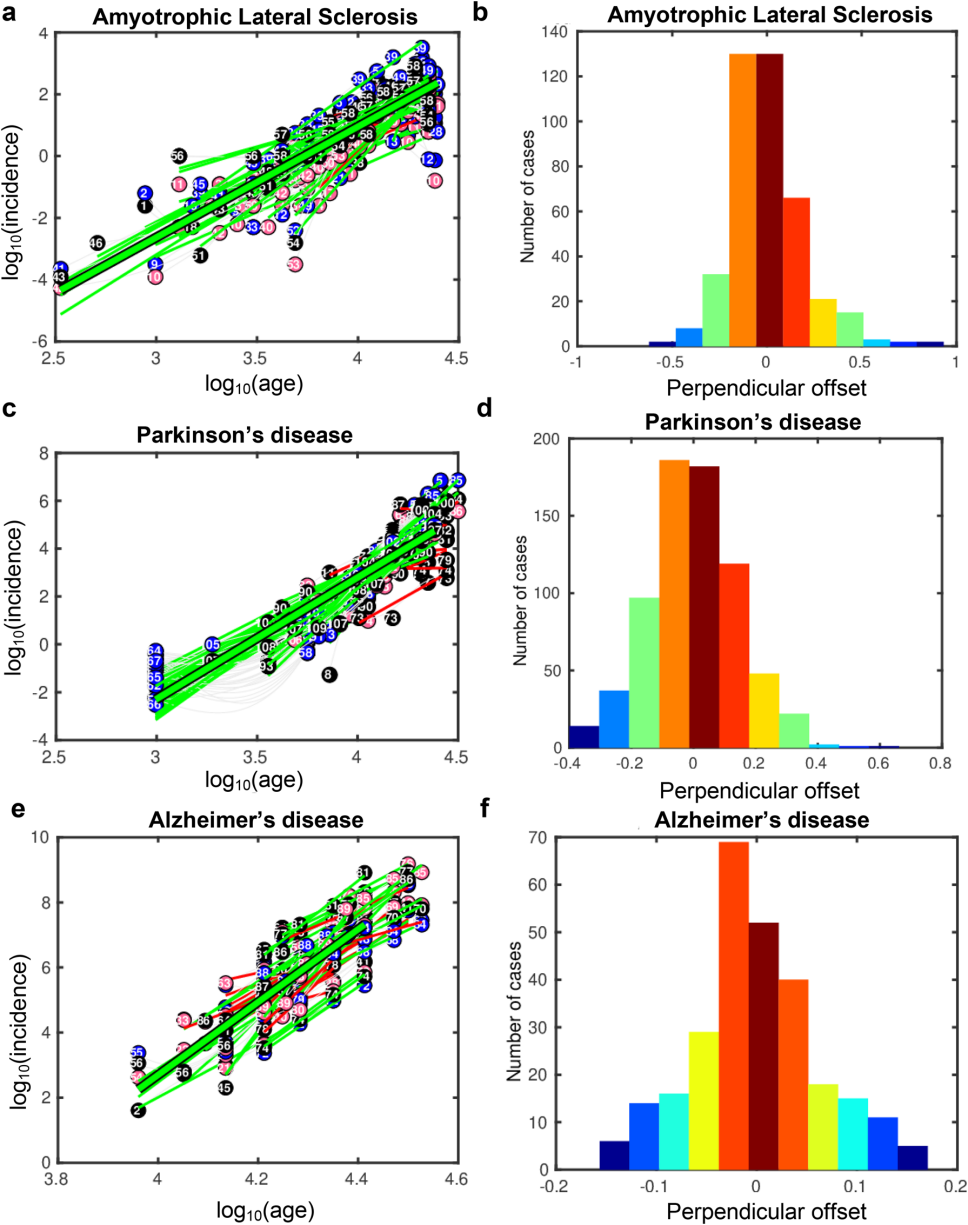
Multistep model analysis of neurodegenerative age-stratified incidence profiles. Regression lines of the fit incidence *versus* age for (**a**) Amyotrophic Lateral Sclerosis, (**c**) Parkinson’s, and (**e**) Alzheimer’s diseases. The black-framed green line is the regression line of the fit to all the datasets. The green and red lines are regression lines of datasets that fit and do not fit the multistep model, respectively. The pink, blue and black circles denote data points from female, male and non-stratified according to sex datasets, respectively. The incidence and age are in log_10_ scale. Histograms of the projection of the data on the orthogonal to the regression line of the datasets for (**b**) Amyotrophic Lateral Sclerosis, (**d**) Parkinson’s, and (**f**) Alzheimer’s diseases.

A high percentage of autopsies show different coexistent proteinopathies in degrees of comparable extent and severity, despite the dominant phenotypic expression shared with clinical criteria of a single nosological entity. Therefore, we hypothesized that there is a mathematical model for the pathogenic process that explains the pattern observed in the NDs. It has been shown that the pattern of incidence of ALS fits well a multistep model^35,39^ prompting the idea of requirement for several successive pathogenic events, called steps, each with a risk or probability for the final occurrence of a disease. We applied this multistep approach to our age-stratified incidence datasets and concluded that most of the ND datasets, with the exception of MS, fit well the multistep model since they follow qualitatively regression lines (Fig. 5a,c,e), the perpendicular offsets to their regression lines follow Gaussian distributions (Fig. 5b,d,f), and they pass the thresholds of the quality assessment metrics *R*^2^ and *p*-val of the F-test of the regression model (Supplementary Table S1).

### A new tree model reveals a pathogenic trunk of common steps and branches of disease-specific events occurring at different ages

Since the typical NDs' incidence patterns span similar age ranges and comply with the multistep model, and considering that the existence of a common pathogenic component for these diseases is under intense discussion, we propose an integral theoretical framework for sharing of steps that serves as a common pathogenic context of the NDs.

We illustrate this theoretical framework with a tree, whose leaves are the NDs. Each ND is positioned at a distance from the stem of the tree equal to the number of steps necessary for the onset of the ND. This distance is walked up the tree trunk and then up the branch segments shared by different NDs until arrival to the specific leaf of each ND. Each segment has the length of one step. The rationale of the tree is that the high similarity of the age-stratified incidence trajectories of two NDs corresponds to high number of common segments; we call these common segments common steps, i.e. the steps predicted by the multistep model for two or more NDs and required for the onset of these NDs.

The algorithm that builds the tree is based on a parsimony approach that searches for common steps between diseases and imposes two conditions to simplify the calculations and the tree: 1) “Preserve the ordinal number of each step” that assumes that the ordinal number of a step in a disease is the same ordinal number of this same step in another disease. 2) “Maximize the number of common steps between diseases” to simplify the tree branches.

**Figure 6:**
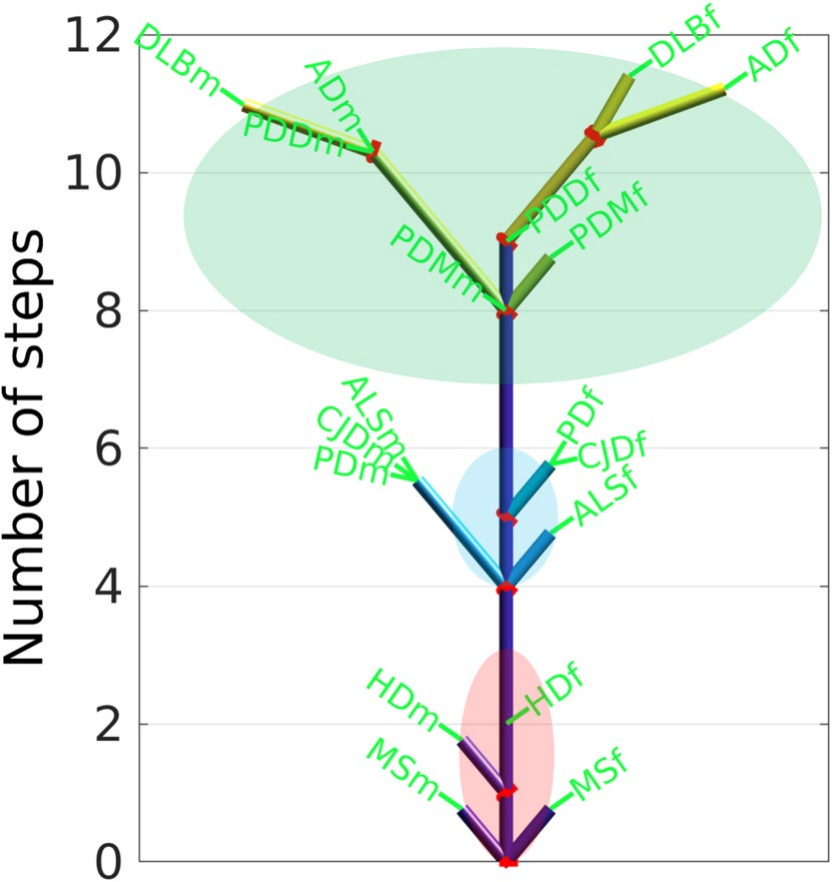
Tree of the genealogy of the neurodegenerative diseases (NDs). The tree shows the number of steps necessary for a ND to occur. The common steps are represented by the trunk of the tree and the non-common, specific steps, by the branches of the tree. The left and right tree sides depict the specific branches of the male and female NDs, NDm and NDf, respectively. The red rings mark the branch-out points. Red, blue and green ellipses mark the stem-, trunk- and crown-associated NDs, respectively. NDs: Amyotrophic Lateral Sclerosis (ALS), Alzheimer's disease (AD), Parkinson's disease (PD), Huntington's disease (HD), Fronto Temporal Dementia (FTD), Dementia with Lewy Bodies (DLB), Parkinsonism (PDM), Parkinson’s Disease with Dementia (PDD), Creutzfeldt–Jakob disease (CJD), Multiple Sclerosis (MS).

We represent the ND multistep model as a tree with a common pathogenic trunk of common steps that drive a core neurodegenerative process, and branches that represent disease-specific steps happening at different ages and producing the different peaks of incidence for each disease (Fig. 6). To reflect the different dynamics of the age-stratified incidence profiles of NDs for male and female, our model considers male and female NDs independently, denoted as NDm and NDf, respectively. The maximum numbers of common steps for male and female NDs are 8 and 9, respectively. MS for male and female, MSm and MSf, do not follow the multistep model, therefore they do not share steps with the other typical NDs. NDs with minimum number of common steps are HDm (1 common step) and HDf (2 common steps), which are purely monogenic NDs. The NDs with the highest number of common steps (11) are AD, DLB and PDD for the male case, and AD and DLB for the female case. For female, AD shows the highest number of steps, 13, with 2 specific steps, while for male DLB has the same highest number of steps, 13, and also with 2 specific steps (Fig. 6). Since there is no stratified data according sex on incidence with age for FTD, we built additionally a tree based on combined data for male and female for all NDs including FTD (Supplementary Fig. S1). We showed that FTD has 6 steps and clusters together with ALS, CJD and PD. When comparing the epidemiological results with the brain autopsies results, it is important to note that FTD is a clinical term; the neuropathological counterpart is Frontotemporal Lobar Degeneration (FTLD) with different causes: TDP-43 proteinopathy, tauopathy or the rarer FTLD (i.e. FUS). For brevity, we used the term FTD to include FTLD-TDP43, FTLD-τ, FLTD-FUS. Additionally, PDM is a clinical term, not neuropathological one, therefore it does not appear in the brain autopsies study.

## Discussion

The number of tree steps is related to the rate of progression of a disease and not the prevalence, thus cases with the same number of steps could have different prevalence, and vice versa. The higher number of steps of an ND is accompanied by a higher number of these steps shared with other NDs. E.g., in DLB and AD, the number of steps required for reaching clinical expression is much higher than those required for a high-penetrance genetic disease such as HD.

The tree has three levels: The stem proximal level with a non-step disease like MS, and a purely genetic disease like HD. The middle trunk level with the cluster of ALS, PD, and CJD; And the crown with AD, DLB, and the Parkinson-associated diseases—PDD and PDM. These ND groups correspond to the ones found by the non-parametric PCA analysis (Fig. 3d), where the red, green and blue ellipses mark the NDs associated to the three levels of the tree—stem, trunk and crown, respectively. Interestingly, the PCA distinguishes between the non-step MS and the purely monogenic HD, both part of the tree stem.

The first, stem branches are the male and female MS which do not follow the multistep model and do not share steps with other NDs. The first real branch of the tree is that of HD, for which the first step had occurred before birth. The next trunk branches are ALS, PD and CJD; ALS requiring one step less for female (in total six) than for male and same number of six steps for PD and CJD. The differences in the step number in male and female ALS could be due to neuroanatomical differences such as the thickness of the bilateral primary motor cortex, reduced in ALS men and unchanged in women, which may be due to both different susceptibilities to damage and different abilities to repair^40^; men have a greater likelihood of onset in the spinal regions, and women in the bulbar region^41^; men and women differ in their exposures to environmental toxins, biological responses to exogenous toxins, and abilities to repair damage^41^. Males are selectively exposed, or genetically predisposed to be susceptible, to influences like smoking, military service, exercise, electrical exposure, heavy metals and agricultural chemicals^42^. PD and CJD manifest same number of steps, common and specific, for males and females. Incidence and prevalence of PD are 1·5–2 times higher in men than in women; anyway the mean Unified Parkinson's Disease Rating Scale III scores at disease onset were equal for both genders, as was the rate of deterioration^43^. There is no gender predilection for CJD^44^. FTD is in the middle trunk level together with ALS, PD, and CJD (Supplementary Fig. S1).

In the crown, PDD and DLB for female have fewer steps, while PDM and AD manifest higher number of steps in the female case compared to the male one. In the DLB case, female DLB patients have a more rapid disease course, and are more likely to present visual hallucinations^45^. Males have a higher risk than females for neocortical Lewy bodies^46^. Female sex is associated with increased risk of AD development, with impact of pregnancy, menopause, influence of estrogens and hormone therapy on the brain function^47^; Women heterozygous or homozygous for the ε4 allele of the APOE gene are at greater risk of developing AD than men with this allele^48^ and they demonstrate more severe behavioral disinhibition^49^.

In the PCA analysis we had three different groups of age-stratified incidence profiles related to NDs, which is quite similar to the result of the stem, trunk and crown of the tree, with HD (1 step); ALS, CJD and PD (4–6 steps) and PDD, AD and DLB (8–12 steps). FTD would be closer to ALS, CJD and PD (with 5 steps). HD is a monogenic condition in which a single factor, the CAG expansion in HTT is sufficient to cause the disease, and the size of the expanded repeat explains around 60% of the age at onset variation of HD. FTD is generally an early-onset form of dementia, in which 20–40% of the cases are associated with mutations in genes related to monogenic diseases (which would imply again a single-cause disease) and there is a strong relation of FTD and ALS, with some genes leading to both phenotypes in a single family. CJD is related to infective prion protein with abnormal conformation that propagate from cell-to cell, and misfolded α-synuclein, related to PD (but also PDD and DLB) seems to have a prion-like propagation pattern. PDD, AD and DLB are later onset forms of ND, occurring generally in the eighth to ninth decade of life. Considering the quite different pathophysiological processes of CJD and PD and FTD/ALS (but with similar number of steps to onset), one could consider that a lower number of factors with greater weights might be responsible for the disease onset in these conditions, but that such factors could be related to completely different pathways. In other words, the general conclusion of our paper is not related to a common pathways for the onset of NDs, but rather to give clues on how complex (number of steps) the disease onset trigger of different NDs is. Which is quite important in order to highlight that future studies should consider the complexity, expressed by the number of steps, of factors related to disease onset prior deciding to study single factors for multiple steps disease.

Neurodegeneration is a manifold scenario. Clinical, epidemiological and neuropathological data give strong evidence that neurodegenerative processes do not start simultaneously in time and space; they rather begin in specific regions with increased vulnerability and higher propensity to propagate later on. The onset might be initiated outside the central nervous system. Additional factor in neurodegeneration is the socio-environmental context. Each individual is unique with a complex plot of genetics and biography where aging of biological systems is associated with the markers of the disease. It is impossible to generate a general theory of neurodegeneration without assuming epistemologically that it is a process of non-linear causality. While our tree does not specify the nature of the steps or their order, it suggests that a ND research project that does not consider a multistep hypothesis and does not aspire to determine the identity and order of these steps will have difficulties in its generality as a humdrum explanatory theory.

## Conclusions

In this work we asked the question: Are the neurodegenerative diseases (NDs) triggered by steps some of which are common for the NDs? Searching for an answer, we designed an algorithm that checks whether a ND follows a multistep model based on over 400 epidemiological datasets of age-stratified incidence of NDs, finds the number of steps necessary for the onset of each ND, finds the number of common and specific steps across the NDs, and builds a parsimony tree of genealogy of the NDs. Our tree model has a pathogenic trunk formed by common steps, and branches of number of disease-specific events occurring at different ages and producing different peaks of incidence for the specific NDs.

## Methods

### Post-mortem human samples

Post-mortem tissues were obtained from the Institute of Neuropathology Brain Bank (HUB-ICO-IDIBELL Biobank) following the practice and expertise of BrainNet Europe Bank (https://www.brainnet-europe.org/) ‘Network of Excellence’ funded by the European Commission in the sixth Framework Program ‘Life Science’ (LSHM-CT-2004–503,039). All samples were obtained in agreement with ethical standards and legislation defined by the European Union and following the approval of the local ethics committee. Tissues from 1,818 diseased patients with available age information were analyzed in total: 7 brains were from patients aged [0–20) years, 67 aged [20–40) years, 415 aged [40–60) years, 986 aged [60–80) years, and 343 aged [80–100) years. Out of the 1,818 patients, 1,787 patients have associated gender information: 1,138 were male and 649 were female. In the female case: 1 brain was from a patient aged [0–20) years, 23 brains were from patients aged [20–40) years, 133 aged [40–60) years, 351 aged [60–80) years, and 141 aged [80–100) years. In the male case: 5 brains wer from patients aged [0-20) years, 44 aged [20-40) years, 275 aged [40-60) years, 621 aged [60-80) years, and 193 aged [80-100) years.  The neuropathological diagnosis and current protocol for the autopsies in adult donors was as follows: one hemisphere was immediately cut in coronal sections, 1 cm thick, and selected areas of the encephalon were rapidly dissected, frozen on metal plates over dry-ice, placed in individual air-tight plastic bags, and stored at -80ºC until use for biochemical studies. The other hemisphere was fixed by immersion in 4% buffered formalin for 3 weeks for morphological studies. For current neuropathological study, twenty representative brain regions were embedded and paraffin: medulla oblongata; mesencephalon (substantia nigra upper level); pons (locus ceruleus); upper cerebellar vermis; cerebellum and dentate nucleus; anterior hippocampus and parahippocampal gyrus; caudate, putamen, accumbens; frontal cortex area 8; primary visual area and area 19; amygdala; basal nucleus of Meynert, globus pallidus; hypothalamus, mammillary bodies; anterior cingulate cortex; posterior hippocampus; parietal cortex at the level of the splenium; anterior superior and middle temporal gyri; posterior middle and inferior temporal gyri at the level of the geniculate nucleus; medial and anterior thalamic nuclei, subthalamic nucleus; posterior thalamus; olfactory bulb and tract. In addition, cervical spinal, thoracic spinal cord; lumbar spinal cord; saccral spinal cord; spinal ganglia; spinal nerve roots, and hypophysis were also analyzed when available. Sections 4-µm-thick obtained with a sliding microtome, were de-waxed and stained with hematoxylin and eosin, periodic acid-Schiff (PAS), and Klüver-Barrera, or processed for immunohistochemistry for microglia Iba1, glial fibrillary acidic protein (GFAP), β-amyloid, phospho-τ (clone AT8), α-synuclein, TDP-43, αB-crystallin and ubiquitin, using EnVision + System peroxidase (Dako, Agilent, CA, USA), and diaminobenzidine and H_2_O_2_. In addition, 1 cm-thick coronal sections of the frontal lobe at the level of the head of the caudate and putamen were obtained in every case. Blocks were embedded in paraffin, cut at a thickness of 7 µm, de-waxed, and stained with haematoxylin and eosin, and with Klüver-Barrera. Additional blocks from different brain regions were also embedded in paraffin and stored for further studies if needed. Details of the procedures and methodological protocols for current neuropathological studies are described elsewhere^50^. Cases and diagnoses are anonymized at the HUB-ICO-IDIBELL biobank.

### Algorithm of the calculation of the genealogy tree of the NDs

#### Calculation of the number of steps of each ND with the multistep regression model

Firstly, for each dataset we fitted the regression equation for each ND incidence *versus* the age profile. As in Armitage & Doll^34^ we used a logarithmic transformation of the regression equation to check whether the pathogenesis of a disease follows a multistep model. The rationale of this transformation is that age and incidence in logarithmic scale must fit a linear regression, and the slope of the regression is directly linked to the average number of steps = slope + 1. The incidence rate is the number of new cases per population at risk in a time period. For a multistep model, the incidence *i* across time *t* is *i* = *u*_1_⋅*u*_2_⋅*u*_3_⋅…*u*_*n*−1_⋅*u*_*n*_⋅*t*^(*n*−1)^, where *u*_*k*_ is the average background risk of step *k.* The regression line in logarithmic scale of *i* across *t* is log(*i*) = (*n* − 1)⋅log(*t*) + *c,* where *n* − 1 = *m* is the slope of the regression line, *n* = *m* + 1 is the number of steps, and *c* = *log*(*u*_1_*⋅u*_2_*⋅u*_3_*⋅*… *u*_*n−1*_*⋅u*_*n*_) = *log*(*u*) is the intercept of the regression line. The background risk *u* of all steps is *c* = log(*u*_1_*⋅u*_2_*⋅u*_3_⋅…*u*_*n*−1_*⋅u*_*n*_) = log(*u*), *u* = exp(*c*). The geometric average background risk of all steps is μ(*u*) = *u*^(1/*n*)^. To fit the regression model, the data corresponding to ages equal or greater than 80 years were truncated under the condition of at least 4 data points remaining.

#### Assessment of the quality of the regression model: R^2^, p-val, Valid

We used the following statistics to assess the statistical significance of the linear regression relationship between the response variable and the predictor variables: *R*^*2*^ coefficient of determination, *p*-val for the F-test on the regression model. If *p*-val < 0.05, the multistep model is considered valid and the “Valid” flag is set to “Y” (yes), otherwise it is set to “N” (no), see Supplementary Table S1.

#### Integrative analysis of the trajectories of incidence versus age of the NDs

To adjust the epidemiological data studies to same age intervals, we modeled the age-stratified incidence trajectories of each study with cubic splines and interpolated each trajectory at the same age points for all datasets. We averaged the incidence trajectories of the different studies corresponding to the same ND *i*, and built a *d* × *a* incidence matrix *I*, where *d* and *a* are the number of NDs and age points, respectively. The element $I\left( {i,j} \right)$ denotes the incidence of disease *i* at age *j*.

#### Calculation of the tree of the genealogy of NDs

**Figure 7:**
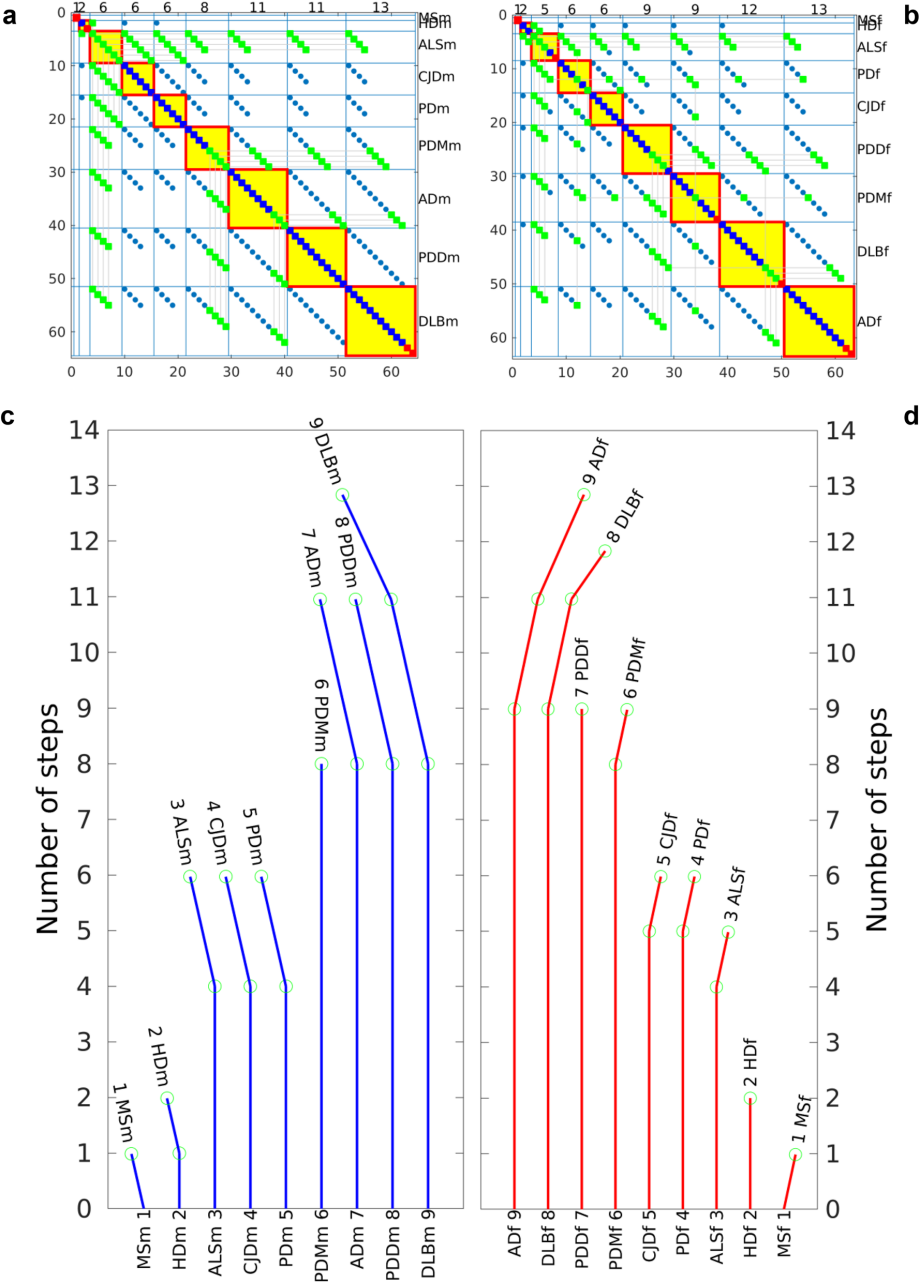
Calculation of the tree of genealogy of neurodegenerative diseases (NDs). Spy of the adjacency matrix *A* before sorting for: (**a**) male and (**b**) female. Blue squares mark the steps in general. The green squares denote promoters of common steps. The promoters of common steps are marked only in the first disease where the step is found to be common but not in the remaining diseases with this common step. Promoter is the first common step found. Red squares mark the non-common steps. The red-bordered yellow squares mark the spy of the matrices used to position each ND on the tree of the genealogy of NDs. Number of steps and deflections calculated for each ND for: (**c**) male and (**d**) female.

1. Calculate a similarity matrix *Sim*(*d* × *d*) providing a measure of similarity of the profiles of the age-stratified incidence using the non-negative Pearson’s correlation coefficient $\rho \left( {i,j} \right) = max\left( {\frac{{cov\left( {I\left( i \right),I\left( j \right)} \right)}}{{\sqrt {cov\left( {I\left( i \right),I\left( i \right)} \right)cov\left( {I\left( j \right),I\left( j \right)} \right)} }},0} \right)$ between the age-stratified incidence trajectories, where *cov*(*I*(*i*),*I*(*j*)) is the covariance between the average age-stratified incidence trajectories *I*(*i*) and *I*(*j*) of diseases *i* and *j*, respectively.
2. Build a matrix of the number of common steps between pairs on NDs *S*(*d* × *d*) by multiplying each element ρ(*i*, *j*) of the similarity matrix *Sim* with the minimal number of steps shared by two diseases, $S\left( {i,j} \right) = \rho \left( {i,j} \right) \times {\min}\left( {n_{i} ,n_{j} } \right)$, where *n*_*i*_ is the number of steps of disease *i*. We define a common step between two NDs as a step predicted by the multistep model of each of the two NDs and shared by the two NDs. $S\left( {i,j} \right)$ is the number of common steps between diseases *i* and *j*. Next, sort the rows and the columns of *S* in increasing order of its diagonal elements.
3. Build a Boolean symmetric adjacency matrix *A*(*n* × *n*) to find which steps are common for which NDs, where *n* is the upper limit of the number of non-common, or specific, steps (Fig. 7a,b). If no pair of NDs had common steps, the maximum number of steps of all NDs would be $n = \mathop \sum \limits_{i = 1}^{d} n_{i}$. If a common step between two diseases has different ordinal number in the two diseases, the possible combinations of the order of common steps between the two diseases would be $\left( { n^{2} - n} \right)/2$. To reduce the search space of common-step combinations, we use a parsimony approach imposing a “*preserving the ordinal number of each step*” criterion, assuming that the ordinal number of a step in a disease is the same ordinal number of this same step in another disease. The matrix of adjacency *A* stores all possible combinations in which a step might be shared by a set of diseases. *A*(*i*_*k*_*, j*_*l*_) indicates whether a step *k* of a disease *i* is the same step *l* of a disease *j*. *A* is a matrix of *d* × *d* blocks *B*_*ij*_, where each *B*_*ij*_ stores the potentially common steps between diseases *i* and *j*. $A(k \in B_{ij} ,\quad l \in B_{ij} )$ is 1, if step *k* of disease *i* is the same step as step *l* of disease *j. B*_*ij*_ is the *n*_i_ × *n*_*j*_ Boolean matrix of the common steps between diseases *i* and *j*. The adjacency matrix *A* is built as follows: First, initialize *A* with zeros. Next, scan the matrix of common steps *S* and set to 1 all the elements of *A* fulfilling the condition $A\left( {k \in B_{ij} ,l \in B_{ij} } \right) = 1$, for 1 < *k* < *S*(*i*,*j*) and 1 < *l* < *S*(*i*,*j*). Each row of *A* is associated to a step *k* across all diseases and indicates the possible cases, marked with 1, in which such step is shared by other diseases. Among all potential common steps, we choose those with higher plausibility to be common among more diseases, introducing “*maximizing the number of shared steps between diseases*” criterion.
4. Calculate a Boolean matrix *D*(*f* × *n*) of branch deflections of the tree of steps, where the number of rows *f* of *D* is the maximum number of deflections determined by the algorithm that builds *D*. Our algorithm selects all possible combinations of steps of *A* with maximum number of sharing. First, it sorts the rows of *A* in descending order of row density. We define as row density the number of ones in a Boolean row. Second, it selects as a first state the row with maximum density and then scans the reordered matrix *A* to choose as new deflections the rows without common steps with all previously created deflections. $D\left( {i,j_{l} } \right) = 1$, if step *l* of disease *j* has a deflection at position *i* of the tree. The algorithm recovers unassigned steps, searching for and creating new branch deflections with them (Fig. 7c,d).

#### Median age of onset of each ND

As additional parameters to address the behavior of the multistep model for each ND dataset, we calculated the median age of onset, the maximum incidence and the age of maximum incidence of each ND, and tabulated them in Supplementary Table S1. The median age of onset for each dataset is estimated as:

$${\text{Age}}\,{\text{onset}} = \frac{{\sum\nolimits_{i = 1}^{n} {\frac{{age_{i}^{Max} - age_{i}^{Min} }}{2} \cdot incidence_{i} } }}{{\sum\nolimits_{i = 1}^{n} {incidence_{i} } }}$$

where *n* is the number of sampling ranges in the dataset, $age_{i}^{Max}$ and $age_{i}^{Min}$ are the maximum and the minimum age defining each sampling range *i*, respectively, $incidence_{i}$ is the incidence in the sampling range *i*.

#### Maximum incidence and age of maximum incidence of each ND

The maximum incidence *incidence*_*Max*_ for the ND of each dataset is the incidence of the sampling range *i*_*Max*_ with maximum incidence across all the sampling ranges in the dataset. The age of maximum incidence *Age incidence*_*Max*_ is the mean of the limits of the age range *i*_*Max*_ of maximum incidence:

$$Age\,incidence_{Max} = \frac{{age_{{i_{Max} }}^{Max} - age_{{i_{Max} }}^{Min} }}{2}.$$

## Supplementary information

Supplementary Information
