# Supplementary material for: Genealogy of the neurodegenerative diseases based on a meta-analysis of age-stratified incidence data
Source: Sci Rep. 2020 Nov 3;10:18923. doi: 10.1038/s41598-020-75014-8 (PMC7609593; doi:10.1038/s41598-020-75014-8)
Supplement: Supplementary file 1 — Supplementary Information [file 41598_2020_75014_MOESM1_ESM.docx]

**Genealogy of the neurodegenerative diseases**

**based on a meta-analysis of age-stratified incidence data**

Daniela Gerovska, PhD^1,2^, Haritz Irizar, PhD^1,3,4^, David Otaegi, PhD^5^, Isidre Ferrer, MD^6^, Adolfo López de Munain, MD^5^, Marcos J. Araúzo-Bravo, PhD^1,2,7,8,9*^

^1^Computational Biology and Systems Biomedicine Group, Biodonostia Health Research Institute, Calle Doctor Beguiristain s/n, 20014 San Sebastián, Spain

^2^Computational Biomedicine Data Analysis Platform, Biodonostia Health Research Institute, Calle Doctor Beguiristain s/n, 20014 San Sebastián, Spain

^3^Icahn Institute for Genomics & Multiscale Biology and Department of Genetics and Genomic Sciences, Icahn School of Medicine at Mount Sinai, New York, NY 10029, United States

^4^Division of Psychiatry, Faculty of Brain Sciences, University College London, WC1E 6BT, London, United Kingdom

^5^Instituto Biodonostia-Hospital Universitario Donostia, San Sebastián, Gipuzkoa, Spain.

^6^Departamento de Patología y Terapéutica Experimental, Universidad de Barcelona, CIBERNED, Hospitalet de LLobregat

^7^IKERBASQUE, Basque Foundation for Science, Calle María Díaz Harokoa 3, 48013 Bilbao, Spain

^8^CIBER of Frailty and Healthy Aging (CIBERfes), Madrid, Spain.

^9^Computational Biology and Bioinformatics Group, Max Planck Institute for Molecular Biomedicine, Röntgenstr. 20, 48149 Münster, Germany

^*^Corresponding author: Marcos J. Araúzo-Bravo: Tel.: +34 943 00 6108, email: [mararabra@yahoo.co.uk](mailto:mararabra@yahoo.co.uk)

**Supplementary Material**

**SUPPLEMENTARY FIGURES**


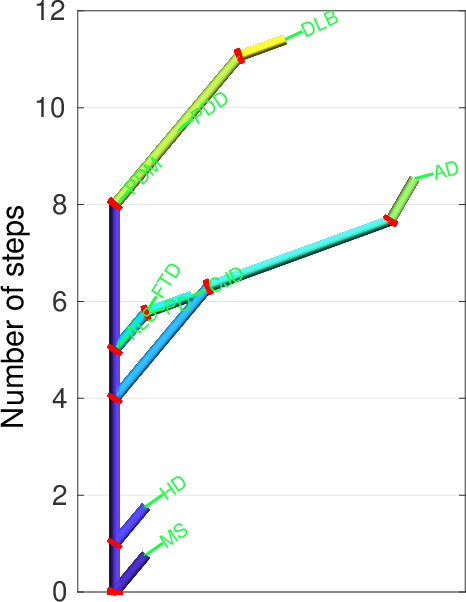


**Figure S1. Tree of the genealogy of the neurodegenerative diseases (NDs) with non-stratified according to sex data.** The tree shows the number of steps necessary for a ND to occur. The common steps are represented by the trunk of the tree and the non-common, specific steps by the branches of the tree. The red rings mark the branch-out points. Red, blue and green ellipses mark the stem-, trunk- and crown-associated NDs, respectively.


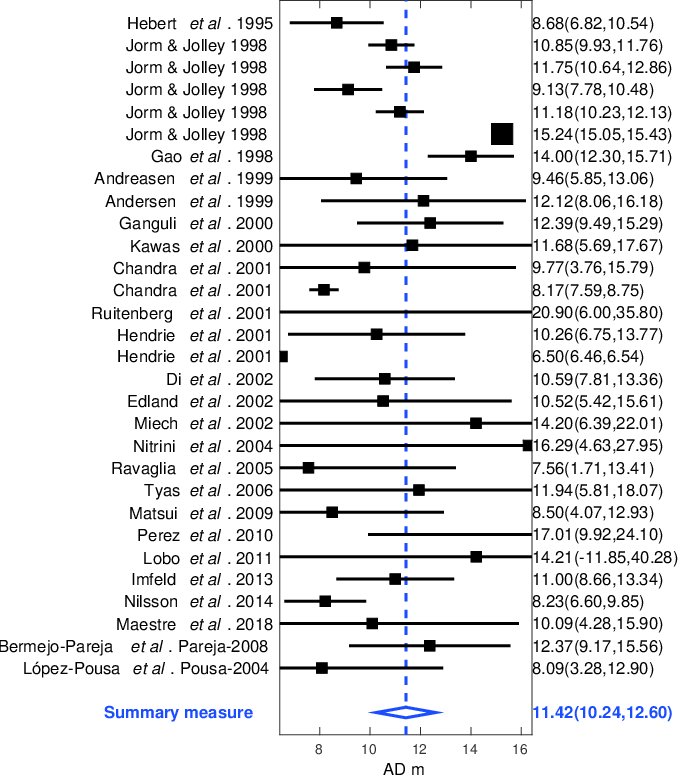


**Figure S2. Forest plot of *m* of disease AD**. The right-hand column is the *m* for each study represented by a square and the confidence intervals represented by horizontal lines. The area of each square is proportional to the weight of the study in the meta-analysis. The overall meta-analyzed *m* is represented as a dashed vertical line. The meta-analyzed *m* is plotted as a diamond, the lateral points of which indicate confidence intervals for this estimate.


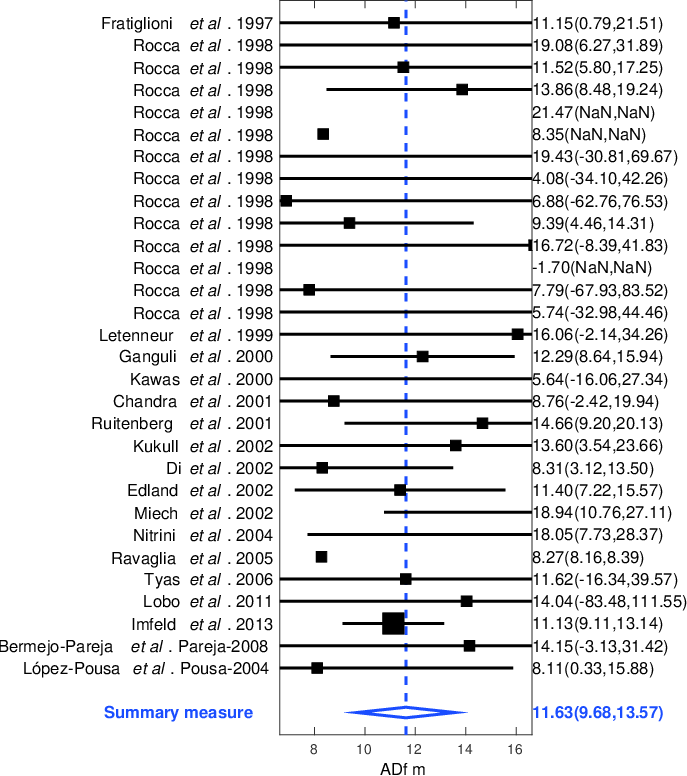


**Figure S3. Forest plot of *m* of disease ADf**. The right-hand column is the *m* for each study represented by a square and the confidence intervals represented by horizontal lines. The area of each square is proportional to the weight of the study in the meta-analysis. The overall meta-analyzed *m* is represented as a dashed vertical line. The meta-analyzed *m* is plotted as a diamond, the lateral points of which indicate confidence intervals for this estimate.


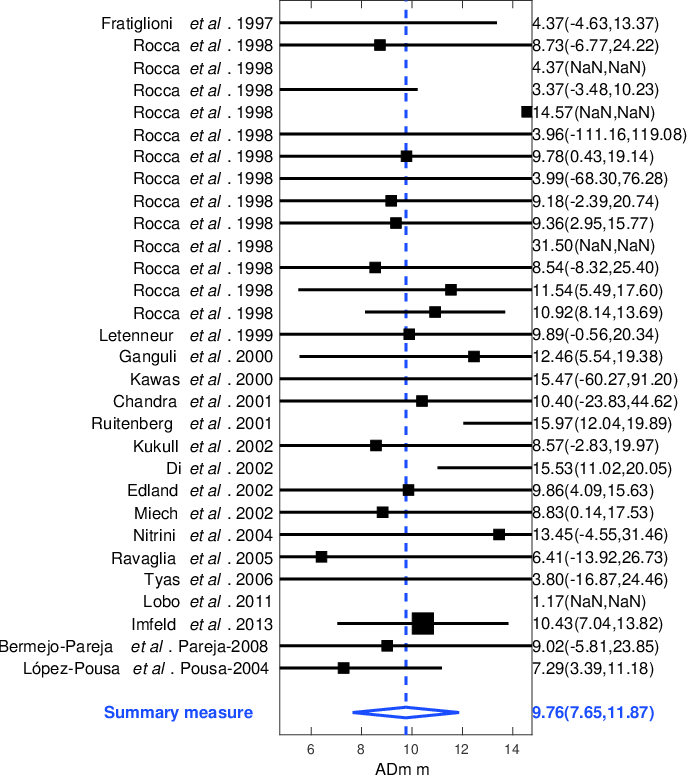


**Figure S4. Forest plot of *m* of disease ADm**. The right-hand column is the *m* for each study represented by a square and the confidence intervals represented by horizontal lines. The area of each square is proportional to the weight of the study in the meta-analysis. The overall meta-analyzed *m* is represented as a dashed vertical line. The meta-analyzed *m* is plotted as a diamond, the lateral points of which indicate confidence intervals for this estimate.


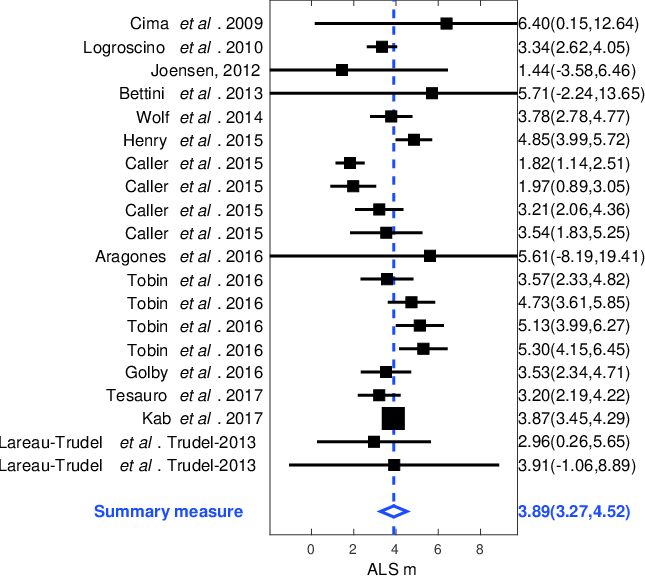


**Figure S5. Forest plot of *m* of disease ALS**. The right-hand column is the *m* for each study represented by a square and the confidence intervals represented by horizontal lines. The area of each square is proportional to the weight of the study in the meta-analysis. The overall meta-analyzed *m* is represented as a dashed vertical line. The meta-analyzed *m* is plotted as a diamond, the lateral points of which indicate confidence intervals for this estimate.


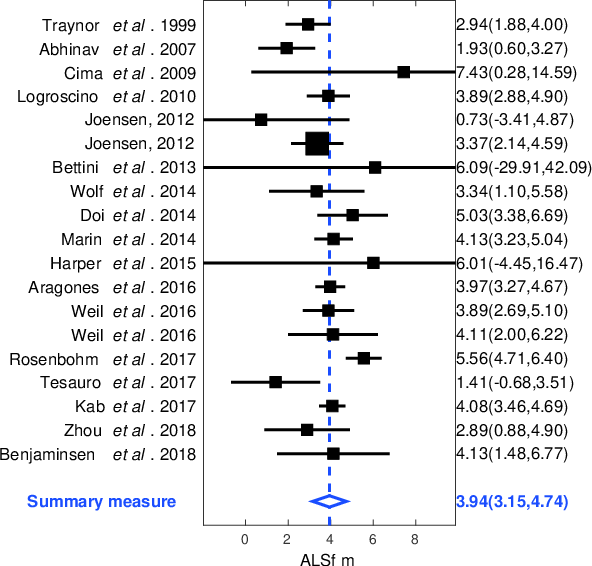


**Figure S6. Forest plot of *m* of disease ALSf**. The right-hand column is the *m* for each study represented by a square and the confidence intervals represented by horizontal lines. The area of each square is proportional to the weight of the study in the meta-analysis. The overall meta-analyzed *m* is represented as a dashed vertical line. The meta-analyzed *m* is plotted as a diamond, the lateral points of which indicate confidence intervals for this estimate.


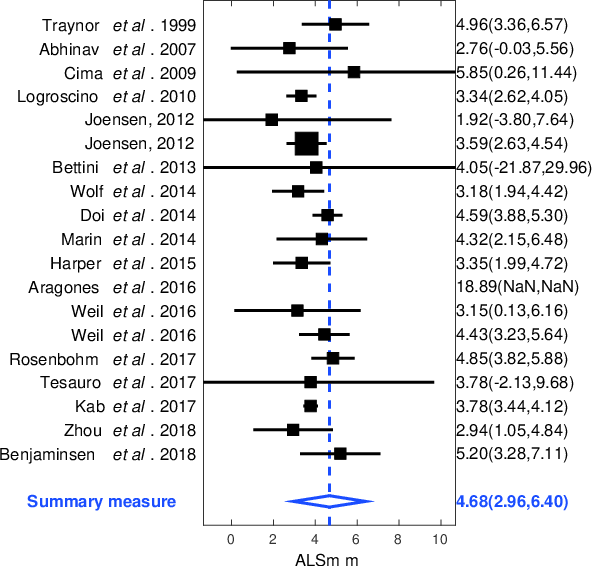


**Figure S7. Forest plot of *m* of disease ALSm**. The right-hand column is the *m* for each study represented by a square and the confidence intervals represented by horizontal lines. The area of each square is proportional to the weight of the study in the meta-analysis. The overall meta-analyzed *m* is represented as a dashed vertical line. The meta-analyzed *m* is plotted as a diamond, the lateral points of which indicate confidence intervals for this estimate.


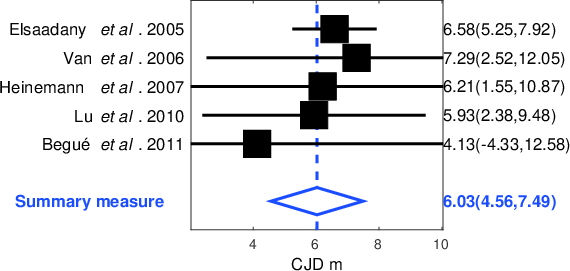


**Figure S8. Forest plot of *m* of disease CJD.** The right-hand column is the *m* for each study represented by a square and the confidence intervals represented by horizontal lines. The area of each square is proportional to the weight of the study in the meta-analysis. The overall meta-analyzed *m* is represented as a dashed vertical line. The meta-analyzed *m* is plotted as a diamond, the lateral points of which indicate confidence intervals for this estimate.


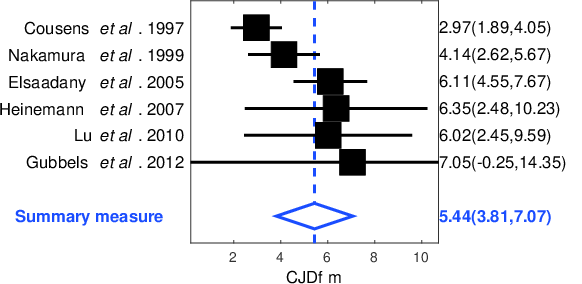


**Figure S9. Forest plot of *m* of disease CJDf**. The right-hand column is the *m* for each study represented by a square and the confidence intervals represented by horizontal lines. The area of each square is proportional to the weight of the study in the meta-analysis. The overall meta-analyzed *m* is represented as a dashed vertical line. The meta-analyzed *m* is plotted as a diamond, the lateral points of which indicate confidence intervals for this estimate.


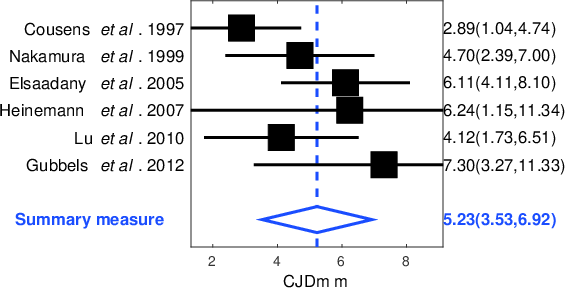


**Figure S10. Forest plot of *m* of disease CJDm**. The right-hand column is the *m* for each study represented by a square and the confidence intervals represented by horizontal lines. The area of each square is proportional to the weight of the study in the meta-analysis. The overall meta-analyzed *m* is represented as a dashed vertical line. The meta-analyzed *m* is plotted as a diamond, the lateral points of which indicate confidence intervals for this estimate.


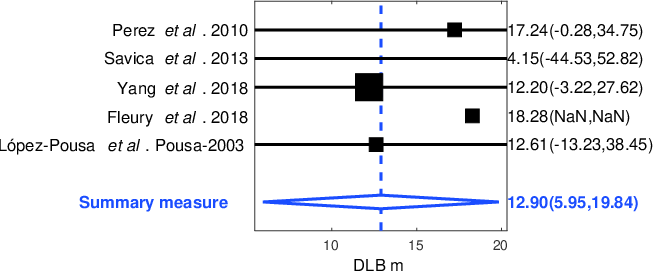


**Figure S11. Forest plot of *m* of disease DLB**. The right-hand column is the *m* for each study represented by a square and the confidence intervals represented by horizontal lines. The area of each square is proportional to the weight of the study in the meta-analysis. The overall meta-analyzed *m* is represented as a dashed vertical line. The meta-analyzed *m* is plotted as a diamond, the lateral points of which indicate confidence intervals for this estimate.


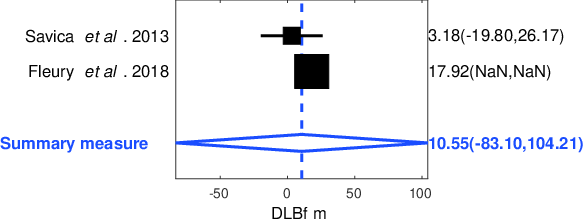


**Figure S12. Forest plot of *m* of disease DLBf**. The right-hand column is the *m* for each study represented by a square and the confidence intervals represented by horizontal lines. The area of each square is proportional to the weight of the study in the meta-analysis. The overall meta-analyzed *m* is represented as a dashed vertical line. The meta-analyzed *m* is plotted as a diamond, the lateral points of which indicate confidence intervals for this estimate.


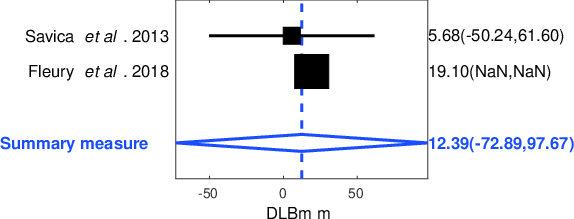


**Figure S13. Forest plot of *m* of disease DLBm**. The right-hand column is the *m* for each study represented by a square and the confidence intervals represented by horizontal lines. The area of each square is proportional to the weight of the study in the meta-analysis. The overall meta-analyzed *m* is represented as a dashed vertical line. The meta-analyzed *m* is plotted as a diamond, the lateral points of which indicate confidence intervals for this estimate.


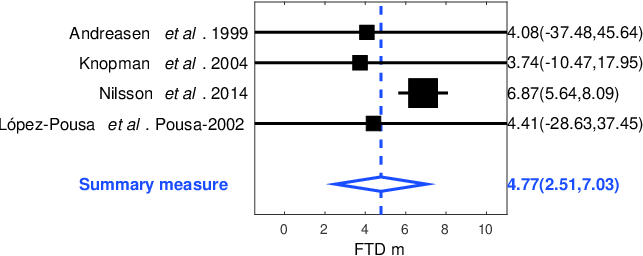


**Figure S14. Forest plot of *m* of disease FTD**. The right-hand column is the *m* for each study represented by a square and the confidence intervals represented by horizontal lines. The area of each square is proportional to the weight of the study in the meta-analysis. The overall meta-analyzed *m* is represented as a dashed vertical line. The meta-analyzed *m* is plotted as a diamond, the lateral points of which indicate confidence intervals for this estimate.


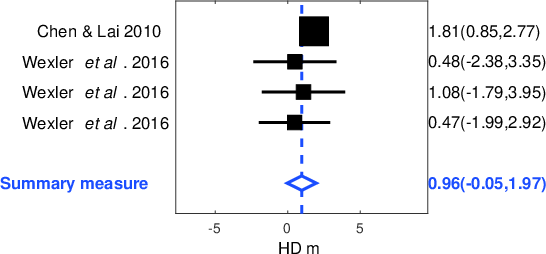


**Figure S15. Forest plot of *m* of disease HD**. The right-hand column is the *m* for each study represented by a square and the confidence intervals represented by horizontal lines. The area of each square is proportional to the weight of the study in the meta-analysis. The overall meta-analyzed *m* is represented as a dashed vertical line. The meta-analyzed *m* is plotted as a diamond, the lateral points of which indicate confidence intervals for this estimate.


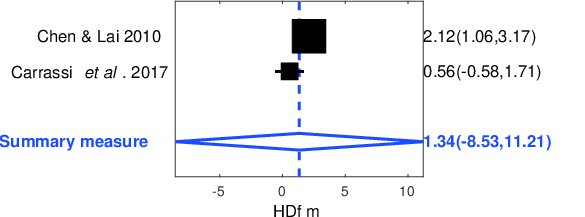


**Figure S16. Forest plot of m of disease HDf**. The right-hand column is the m for each study represented by a square and the confidence intervals represented by horizontal lines. The area of each square is proportional to the weight of the study in the meta-analysis. The overall meta-analyzed m is represented as a dashed vertical line. The meta-analyzed m is plotted as a diamond, the lateral points of which indicate confidence intervals for this estimate.


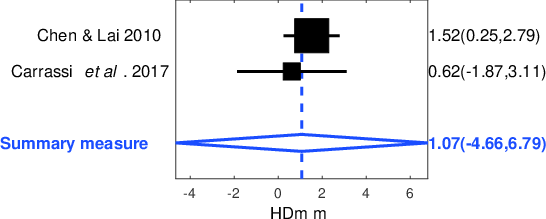


**Figure S17. Forest plot of *m* of disease HDm**. The right-hand column is the *m* for each study represented by a square and the confidence intervals represented by horizontal lines. The area of each square is proportional to the weight of the study in the meta-analysis. The overall meta-analyzed *m* is represented as a dashed vertical line. The meta-analyzed *m* is plotted as a diamond, the lateral points of which indicate confidence intervals for this estimate.


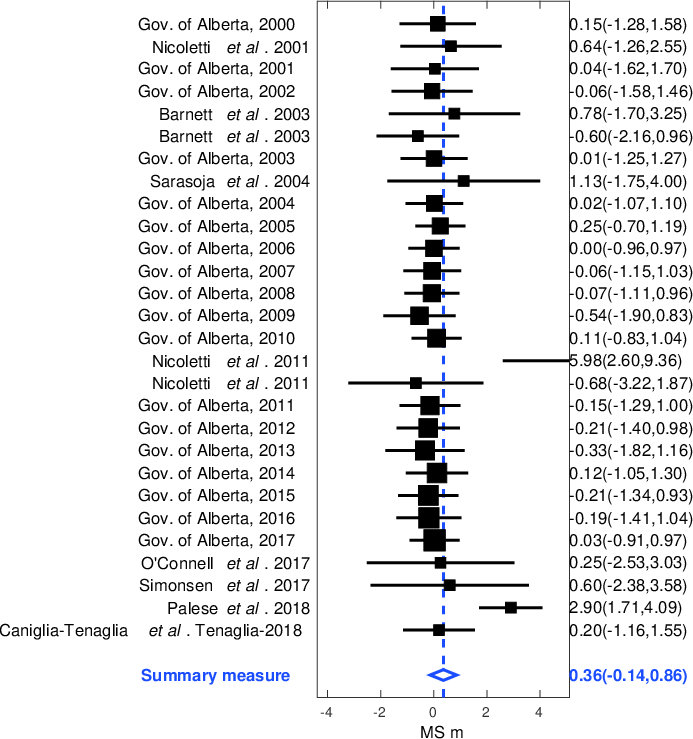


**Figure S18. Forest plot of *m* of disease MS**. The right-hand column is the *m* for each study represented by a square and the confidence intervals represented by horizontal lines. The area of each square is proportional to the weight of the study in the meta-analysis. The overall meta-analyzed *m* is represented as a dashed vertical line. The meta-analyzed *m* is plotted as a diamond, the lateral points of which indicate confidence intervals for this estimate.


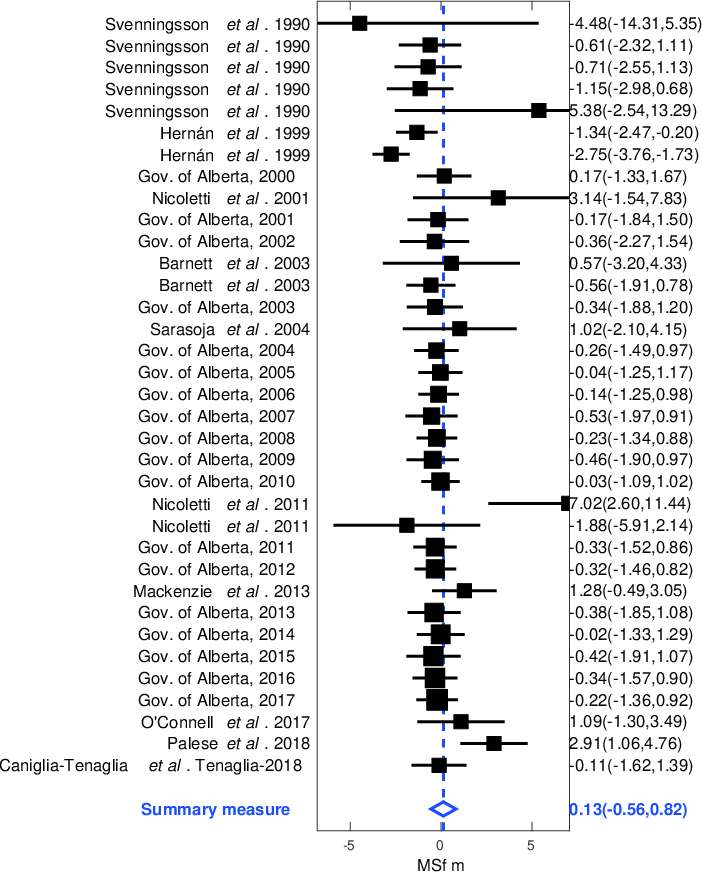


**Figure S19 Forest plot of *m* of disease MSf**. The right-hand column is the *m* for each study represented by a square and the confidence intervals represented by horizontal lines. The area of each square is proportional to the weight of the study in the meta-analysis. The overall meta-analyzed *m* is represented as a dashed vertical line. The meta-analyzed *m* is plotted as a diamond, the lateral points of which indicate confidence intervals for this estimate.


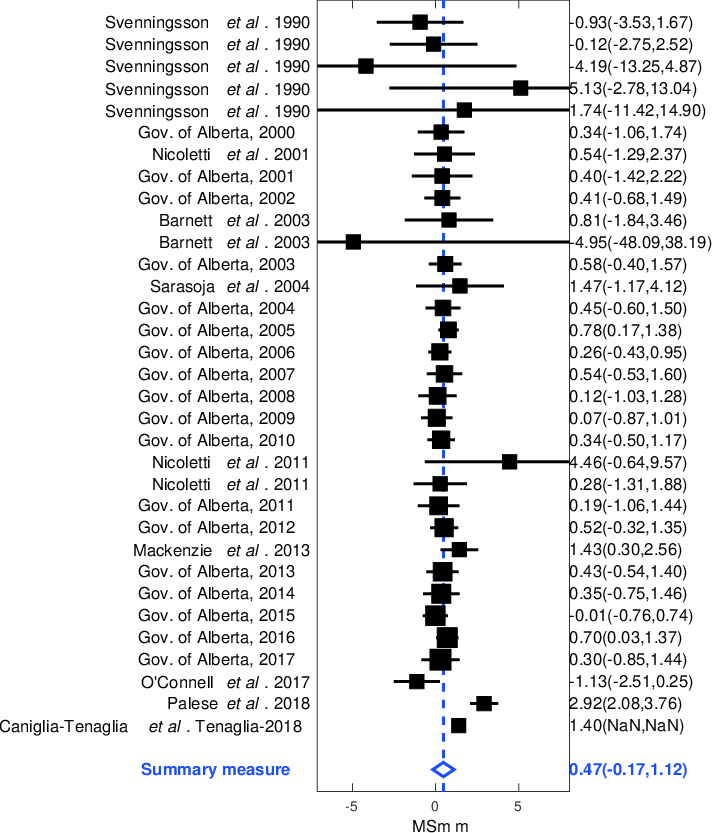


**Figure S20. Forest plot of *m* of disease MSm.** The right-hand column is the *m* for each study represented by a square and the confidence intervals represented by horizontal lines. The area of each square is proportional to the weight of the study in the meta-analysis. The overall meta-analyzed *m* is represented as a dashed vertical line. The meta-analyzed *m* is plotted as a diamond, the lateral points of which indicate confidence intervals for this estimate.


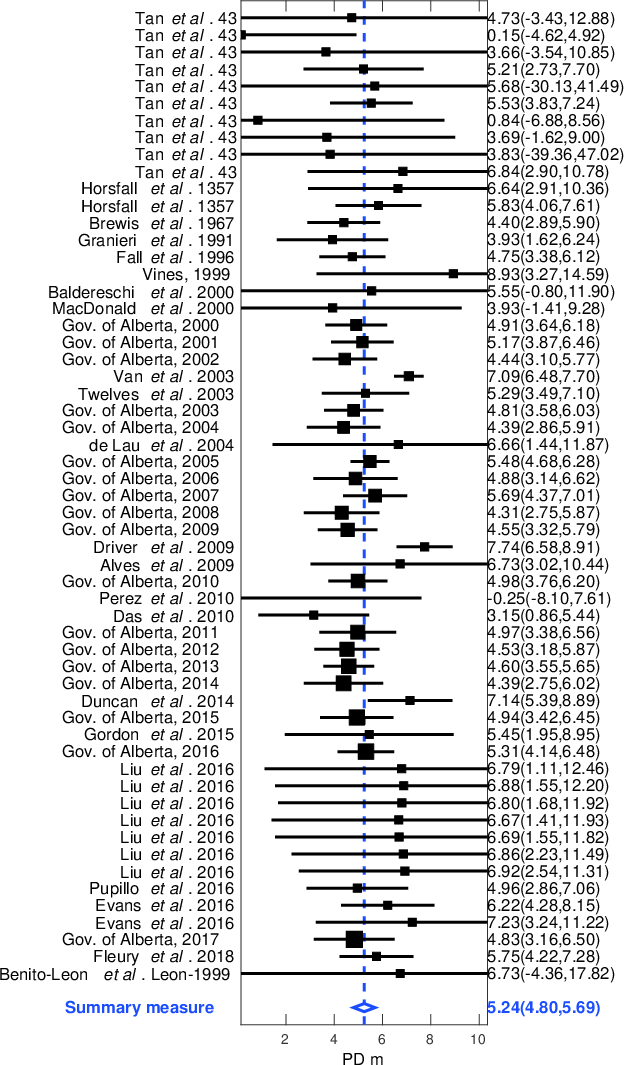


**Figure S21. Forest plot of *m* of disease PD**. The right-hand column is the *m* for each study represented by a square and the confidence intervals represented by horizontal lines. The area of each square is proportional to the weight of the study in the meta-analysis. The overall meta-analyzed *m* is represented as a dashed vertical line. The meta-analyzed *m* is plotted as a diamond, the lateral points of which indicate confidence intervals for this estimate.


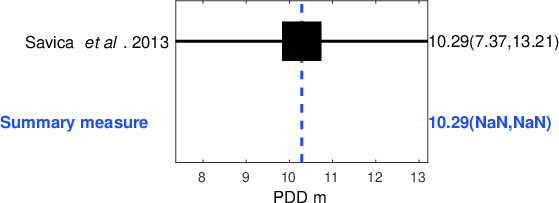


**Figure S22. Forest plot of *m* of disease PDD**. The right-hand column is the *m* for each study represented by a square and the confidence intervals represented by horizontal lines. The area of each square is proportional to the weight of the study in the meta-analysis. The overall meta-analyzed *m* is represented as a dashed vertical line. The meta-analyzed *m* is plotted as a diamond, the lateral points of which indicate confidence intervals for this estimate.


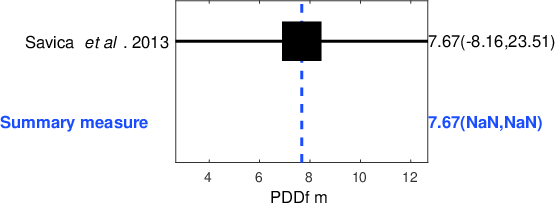


**Figure S23. Forest plot of *m* of disease PDDf.** The right-hand column is the *m* for each study represented by a square and the confidence intervals represented by horizontal lines. The area of each square is proportional to the weight of the study in the meta-analysis. The overall meta-analyzed *m* is represented as a dashed vertical line. The meta-analyzed *m* is plotted as a diamond, the lateral points of which indicate confidence intervals for this estimate.


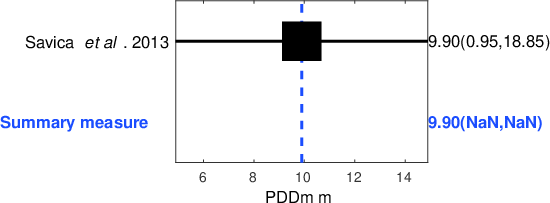


**Figure S24. Forest plot of *m* of disease PDDm**. The right-hand column is the *m* for each study represented by a square and the confidence intervals represented by horizontal lines. The area of each square is proportional to the weight of the study in the meta-analysis. The overall meta-analyzed *m* is represented as a dashed vertical line. The meta-analyzed *m* is plotted as a diamond, the lateral points of which indicate confidence intervals for this estimate.


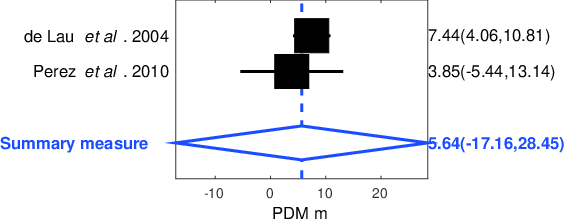


**Figure S25. Forest plot of *m* of disease PDM**. The right-hand column is the *m* for each study represented by a square and the confidence intervals represented by horizontal lines. The area of each square is proportional to the weight of the study in the meta-analysis. The overall meta-analyzed *m* is represented as a dashed vertical line. The meta-analyzed *m* is plotted as a diamond, the lateral points of which indicate confidence intervals for this estimate.


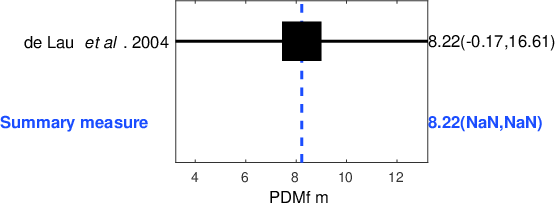


**Figure S26. Forest plot of *m* of disease PDMf**. The right-hand column is the *m* for each study represented by a square and the confidence intervals represented by horizontal lines. The area of each square is proportional to the weight of the study in the meta-analysis. The overall meta-analyzed *m* is represented as a dashed vertical line. The meta-analyzed *m* is plotted as a diamond, the lateral points of which indicate confidence intervals for this estimate.


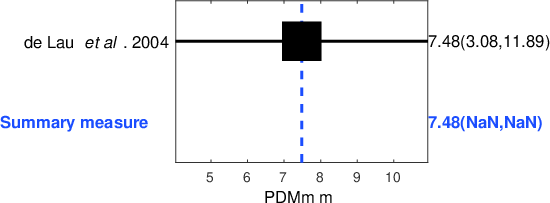


**Figure S27. Forest plot of *m* of disease PDMm**. The right-hand column is the *m* for each study represented by a square and the confidence intervals represented by horizontal lines. The area of each square is proportional to the weight of the study in the meta-analysis. The overall meta-analyzed *m* is represented as a dashed vertical line. The meta-analyzed *m* is plotted as a diamond, the lateral points of which indicate confidence intervals for this estimate.


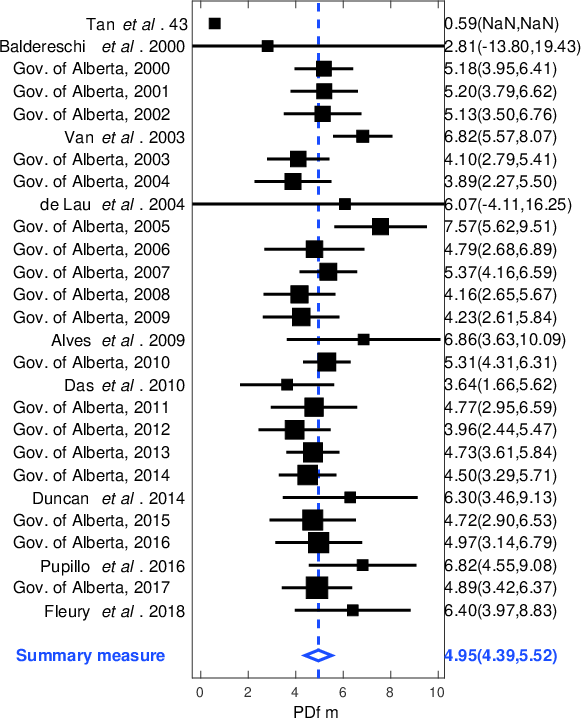


**Figure S28. Forest plot of *m* of disease PDf**. The right-hand column is the *m* for each study represented by a square and the confidence intervals represented by horizontal lines. The area of each square is proportional to the weight of the study in the meta-analysis. The overall meta-analyzed *m* is represented as a dashed vertical line. The meta-analyzed *m* is plotted as a diamond, the lateral points of which indicate confidence intervals for this estimate.


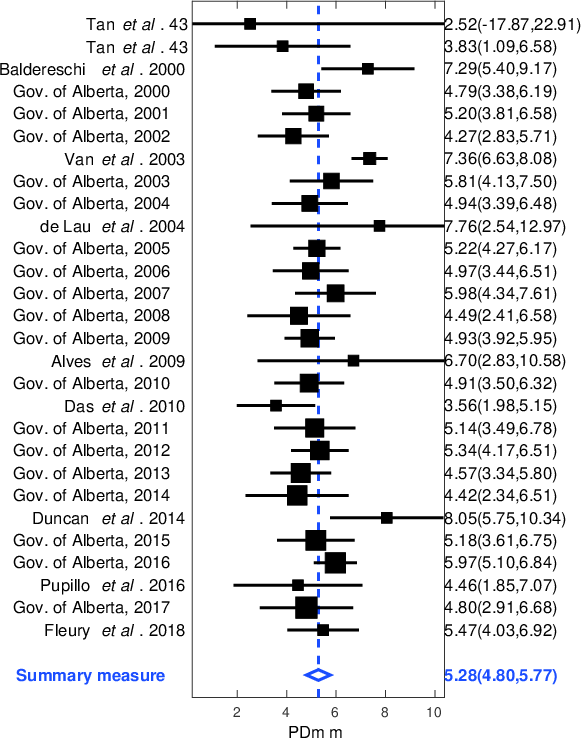


**Figure S29. Forest plot of *m* of disease PDm**. The right-hand column is the *m* for each study represented by a square and the confidence intervals represented by horizontal lines. The area of each square is proportional to the weight of the study in the meta-analysis. The overall meta-analyzed *m* is represented as a dashed vertical line. The meta-analyzed *m* is plotted as a diamond, the lateral points of which indicate confidence intervals for this estimate.

**SUPPLEMENTARY TABLES**

**Table S1. Parameters associated to the multistep model of the NDs for each dataset.** NDx: name of the Neurodegenerative Disease with its sex x: f, m and none for female, male and non-stratified, respectively; Ord.: order of the dataset in each ND; Reference: publication from where the dataset has been collected; Region: the region of each study; Steps: number of steps determined by the multistep model; Risk: risk determined by the multistep model; Age onset: median of the age of onset of the dataset; Max. Inc.: maximum incidence; Age Max. inc.: age of maximum incidence; R^2^: R^2^ statistics of the multistep model; *p*-val: *p* value of the multistep model; Valid: Y, if the multistep model is valid, and N, if the multistep model is not valid.

| **NDx** | **Ord** | **Reference** | **Region** | **Steps** | **Risk** | **Age**  **onset** | **Max**  **inc.** | **Age**  **max.**  **Inc.** | **R^2^** | ***p-*val** | **Valid** |
| --- | --- | --- | --- | --- | --- | --- | --- | --- | --- | --- | --- |
| FTD | 1 | Nilsson *et al*. 201473 | Sweden | 7 | -28 | 69 | 6 | 77 | 0.9615 | 0.0000 | Y |
| FTD | 2 | Knopman *et al*. 200453 | Rochester | 4 | -14 | 59 | 9 | 65 | 0.9180 | 0.1849 | N |
| FTD | 3 | López-Pousa *et al*. 200263 | Girona-UVAMID-1999-2000 | 4 | -15 | 72 | 57 | 77 | 0.7418 | 0.3393 | N |
| FTD | 4 | Andreasen *et al*. 19994 | Piteå River Valley, Sweden-1990-96 | 4 | -14 | 73 | 39 | 77 | 0.6087 | 0.4303 | N |
| DLB | 1 | López-Pousa *et al*. 200362 | Girona | 13 | -51 | 79 | 69 | 82 | 0.6880 | 0.1705 | N |
| DLB | 2 | Savica *et al*. 201384 | Olmsted | 4 | -15 | 77 | 45 | 75 | 0.5394 | 0.4749 | N |
| DLBm | 3 | Savica *et al*. 201384 | Olmsted | 6 | -21 | 77 | 78 | 75 | 0.6250 | 0.4196 | N |
| DLBf | 4 | Savica *et al*. 201384 | Olmsted | 3 | -11 | 77 | 20 | 75 | 0.7558 | 0.3290 | N |
| DLB | 5 | Perez *et al*. 201077 | Gironde-Dordogne,France | 17 | -71 | 80 | 175 | 82 | 0.9936 | 0.0508 | N |
| DLB | 6 | Yang *et al*. 201899 | Taiwan | 12 | -49 | 83 | 122 | 90 | 0.8528 | 0.0765 | N |
| DLB | 7 | Fleury *et al*. 201831 | Geneva, Switzerland | 18 | -76 | 74 | 30 | 75 | 1.0000 | NaN | Y |
| DLBm | 8 | Fleury *et al*. 201831 | Geneva, Switzerland | 19 | -79 | 74 | 35 | 75 | 1.0000 | NaN | Y |
| DLBf | 9 | Fleury *et al*. 201831 | Geneva, Switzerland | 18 | -74 | 74 | 26 | 75 | 1.0000 | NaN | Y |
| ALS | 1 | Logroscino *et al*. 201059 | Europe | 3 | -12 | 64 | 8 | 72 | 0.9061 | 0.0000 | Y |
| ALSm | 2 | Logroscino *et al*. 201059 | Europe | 3 | -12 | 64 | 11 | 72 | 0.9046 | 0.0000 | Y |
| ALSf | 3 | Logroscino *et al*. 201059 | Europe | 4 | -15 | 65 | 7 | 77 | 0.8797 | 0.0000 | Y |
| ALS | 4 | Joensen, 201249 | Faroe Islands-1987-2009 | 1 | -4 | 61 | 12 | 60 | 0.2174 | 0.4286 | N |
| ALSm | 5 | Joensen, 201249 | Faroe Islands-1987-2009 | 2 | -6 | 62 | 16 | 60 | 0.2755 | 0.3638 | N |
| ALSf | 6 | Joensen, 201249 | Faroe Islands-1987-2009 | 1 | -2 | 60 | 8 | 60 | 0.0954 | 0.6131 | N |
| ALSm | 7 | Joensen, 201249 | Europe-2000-1 | 4 | -13 | 67 | 10 | 70 | 0.9645 | 0.0005 | Y |
| ALSf | 8 | Joensen, 201249 | Europe-2000-1 | 3 | -13 | 66 | 7 | 70 | 0.9356 | 0.0016 | Y |
| ALSm | 9 | Zhou *et al*. 2018100 | Beijing 2010-15 | 3 | -12 | 59 | 4 | 60 | 0.7609 | 0.0104 | Y |
| ALSf | 10 | Zhou *et al*. 2018100 | Beijing 2010-15 | 3 | -12 | 59 | 2 | 60 | 0.7329 | 0.0139 | Y |
| ALSf | 11 | Abhinav *et al*. 20071 | SE England | 2 | -7 | 64 | 3 | 62 | 0.6256 | 0.0111 | Y |
| ALSm | 12 | Abhinav *et al*. 20071 | SE England | 3 | -11 | 61 | 4 | 72 | 0.4383 | 0.0521 | N |
| ALSm | 13 | Aragones *et al*. 20165 | Osona, Catalonia, Spain | 19 | -78 | 74 | 24 | 75 | 1.0000 | NaN | Y |
| ALSf | 14 | Aragones *et al*. 20165 | Osona, Catalonia, Spain | 4 | -15 | 69 | 14 | 75 | 0.9998 | 0.0088 | Y |
| ALS | 15 | Aragones *et al*. 20165 | Osona, Catalonia, Spain | 6 | -22 | 71 | 18 | 75 | 0.9639 | 0.1218 | N |
| ALSm | 16 | Wolf *et al*. 201498 | Rheinland-Palatinate-2010-11-Germany | 3 | -12 | 66 | 9 | 72 | 0.8401 | 0.0005 | Y |
| ALSf | 17 | Wolf *et al*. 201498 | Rheinland-Palatinate-2010-11-Germany | 3 | -13 | 66 | 11 | 72 | 0.6406 | 0.0096 | Y |
| ALS | 18 | Wolf *et al*. 201498 | Rheinland-Palatinate-2010-11-Germany | 4 | -15 | 66 | 10 | 72 | 0.9049 | 0.0000 | Y |
| ALSm | 19 | Rosenbohm *et al*. 201781 | Swabia-2012-13-Germany | 5 | -18 | 68 | 14 | 77 | 0.9462 | 0.0000 | Y |
| ALSf | 20 | Rosenbohm *et al*. 201781 | Swabia, Germany-2012-13 | 6 | -22 | 68 | 10 | 77 | 0.9775 | 0.0000 | Y |
| ALSm | 21 | Tesauro *et al*. 201788 | Novara, Italy | 4 | -14 | 70 | 15 | 80 | 0.5802 | 0.1345 | N |
| ALSf | 22 | Tesauro *et al*. 201788 | Novara, Italy | 1 | -5 | 67 | 6 | 80 | 0.8076 | 0.1014 | N |
| ALS | 23 | Tesauro *et al*. 201788 | Novara, Italy | 3 | -12 | 68 | 9 | 80 | 0.9712 | 0.0021 | Y |
| ALS | 24 | Tobin *et al*. 201689 | Ireland-1996-2000 | 4 | -13 | 65 | 13 | 72 | 0.8914 | 0.0004 | Y |
| ALS | 25 | Tobin *et al*. 201689 | Ireland-2001-2005 | 5 | -18 | 67 | 13 | 72 | 0.9469 | 0.0000 | Y |
| ALS | 26 | Tobin *et al*. 201689 | Ireland-2006-2010 | 5 | -19 | 68 | 15 | 77 | 0.9529 | 0.0000 | Y |
| ALS | 27 | Tobin *et al*. 201689 | Ireland-2011-2014 | 5 | -20 | 67 | 15 | 77 | 0.9547 | 0.0000 | Y |
| ALSm | 28 | Weil *et al*. 201696 | Israel, 1997-2005 | 3 | -12 | 65 | 11 | 71 | 0.6773 | 0.0442 | Y |
| ALSm | 29 | Weil *et al*. 201696 | Israel, 2006-2013 | 4 | -17 | 69 | 10 | 71 | 0.9630 | 0.0005 | Y |
| ALSf | 30 | Weil *et al*. 201696 | Israel, 1997-2005 | 4 | -15 | 68 | 7 | 71 | 0.9526 | 0.0009 | Y |
| ALSf | 31 | Weil *et al*. 201696 | Israel, 2006-2013 | 4 | -16 | 68 | 5 | 71 | 0.8796 | 0.0057 | Y |
| ALSf | 32 | Traynor *et al*. 199990 | Ireland, 1995-1997 | 3 | -10 | 65 | 11 | 72 | 0.8601 | 0.0003 | Y |
| ALSm | 33 | Traynor *et al*. 199990 | Ireland, 1995-97 | 5 | -18 | 65 | 13 | 77 | 0.8644 | 0.0001 | Y |
| ALSm | 34 | Benjaminsen *et al*. 201810 | Nordland county, Norway, 2000-15 | 5 | -20 | 68 | 20 | 72 | 0.9068 | 0.0009 | Y |
| ALSf | 35 | Benjaminsen *et al*. 201810 | Nordland county, Norway, 2000-15 | 4 | -16 | 67 | 8 | 72 | 0.7632 | 0.0102 | Y |
| ALSm | 36 | Bettini *et al*. 201312 | Buenos Aires, Argentina-2003-10 | 4 | -15 | 71 | 21 | 75 | 0.7974 | 0.2972 | N |
| ALSf | 37 | Bettini *et al*. 201312 | Buenos Aires-Argentina-2003-10 | 6 | -24 | 69 | 9 | 75 | 0.8221 | 0.2772 | N |
| ALS | 38 | Bettini *et al*. 201312 | Buenos Aires-Argentina-2003-10 | 6 | -22 | 69 | 12 | 75 | 0.8270 | 0.0906 | N |
| ALSm | 39 | Doi *et al*. 201424 | Japan, 2009-10 | 5 | -16 | 66 | 33 | 75 | 0.9877 | 0.0001 | Y |
| ALSf | 40 | Doi *et al*. 201424 | Japan, 2009-10 | 5 | -20 | 66 | 5 | 75 | 0.9689 | 0.0023 | Y |
| ALSm | 41 | Kab *et al*. 201751 | France, 2012-14 | 4 | -14 | 67 | 19 | 77 | 0.9838 | 0.0000 | Y |
| ALSf | 42 | Kab *et al*. 201751 | France, 2012-14 | 4 | -15 | 68 | 13 | 77 | 0.9556 | 0.0000 | Y |
| ALS | 43 | Kab *et al*. 201751 | France, 2012-14 | 4 | -14 | 67 | 15 | 77 | 0.9765 | 0.0000 | Y |
| ALSf | 44 | Harper *et al*. 201541 | Minnesota, 2013-14 | 6 | -24 | 65 | 6 | 65 | 0.7533 | 0.1321 | N |
| ALSm | 45 | Harper *et al*. 201541 | Minnesota, 2013-14 | 3 | -12 | 66 | 13 | 75 | 0.9209 | 0.0024 | Y |
| ALS | 46 | Golby *et al*. 201635 | British Columbia, 2010-15 | 4 | -13 | 66 | 14 | 75 | 0.9445 | 0.0012 | Y |
| ALS | 47 | Lareau-Trudel *et al*. 201355 | Saguenay region, 1985-2004 | 3 | -11 | 66 | 5 | 75 | 0.9949 | 0.0456 | Y |
| ALS | 48 | Lareau-Trudel *et al*. 201355 | Saguenay region, 2005-2009 | 4 | -14 | 69 | 12 | 75 | 0.9901 | 0.0635 | N |
| ALSm | 49 | Marin *et al*. 201467 | Limousin region, 2000-11 | 4 | -16 | 68 | 16 | 77 | 0.7258 | 0.0018 | Y |
| ALSf | 50 | Marin *et al*. 201467 | Limousin region, 2000-11 | 4 | -16 | 66 | 9 | 72 | 0.9325 | 0.0000 | Y |
| ALS | 51 | Henry *et al*. 201545 | New Jersey, 2009-11 | 5 | -19 | 66 | 8 | 75 | 0.9838 | 0.0001 | Y |
| ALSm | 52 | Cima *et al*. 200920 | Padova, Italy, 1992-2005 | 6 | -23 | 66 | 5 | 67 | 0.7871 | 0.0447 | Y |
| ALSf | 53 | Cima *et al*. 200920 | Padova, Italy, 1992-2005 | 7 | -30 | 66 | 7 | 67 | 0.7846 | 0.0456 | Y |
| ALS | 54 | Cima *et al*. 200920 | Padova, Italy, 1992-2005 | 6 | -26 | 66 | 6 | 67 | 0.7800 | 0.0471 | Y |
| ALS | 55 | Caller *et al*. 201515 | New Hampshire, USA-2004 | 2 | -6 | 57 | 6 | 77 | 0.8493 | 0.0004 | Y |
| ALS | 56 | Caller *et al*. 201515 | New Hampshire, USA-2007 | 2 | -7 | 62 | 16 | 72 | 0.6549 | 0.0026 | Y |
| ALS | 57 | Caller *et al*. 201515 | New Hampshire, USA-2005 | 3 | -11 | 65 | 12 | 72 | 0.8619 | 0.0003 | Y |
| ALS | 58 | Caller *et al*. 201515 | New Hampshire, USA-2006 | 4 | -13 | 66 | 19 | 72 | 0.7736 | 0.0018 | Y |
| PDf | 1 | Van *et al*. 200393 | Califonia | 7 | -25 | 70 | 78 | 75 | 0.9902 | 0.0004 | Y |
| PDm | 2 | Van *et al*. 200393 | Califonia | 7 | -27 | 70 | 141 | 75 | 0.9971 | 0.0001 | Y |
| PD | 3 | Van *et al*. 200393 | Califonia | 7 | -26 | 70 | 107 | 75 | 0.9978 | 0.0000 | Y |
| PD | 4 | Baldereschi *et al*. 20006 | Europe | 6 | -18 | 77 | 678 | 82 | 0.8760 | 0.0640 | N |
| PDm | 5 | Baldereschi *et al*. 20006 | Europe | 7 | -25 | 77 | 942 | 82 | 0.9928 | 0.0036 | Y |
| PDf | 6 | Baldereschi *et al*. 20006 | Europe | 3 | -7 | 76 | 419 | 82 | 0.2097 | 0.5420 | N |
| PD | 7 | Granieri *et al*. 199139 | Europe | 4 | -13 | 63 | 44 | 62 | 0.6987 | 0.0050 | Y |
| PD | 8 | Vines, 199995 | Navarra | 9 | -34 | 69 | 52 | 72 | 0.7670 | 0.0098 | Y |
| PD | 9 | Benito-Leon *et al*. 20049 | Central Spain | 7 | -24 | 77 | 298 | 82 | 0.7733 | 0.1206 | N |
| PD | 10 | Fall *et al*. 199630 | Sweden | 5 | -17 | 69 | 59 | 75 | 0.9760 | 0.0016 | Y |
| PD | 11 | MacDonald *et al*. 200064 | UK | 4 | -12 | 70 | 222 | 72 | 0.6467 | 0.1009 | N |
| PD | 12 | Brewis *et al*. 196713 | Europe | 4 | -15 | 67 | 65 | 75 | 0.9875 | 0.0063 | Y |
| PD | 13 | Twelves *et al*. 200391 | World | 5 | -19 | 62 | 45 | 62 | 0.8957 | 0.0004 | Y |
| PD | 14 | CPRD„ 201714 | UK | 8 | -28 | 71 | 174 | 77 | 0.9995 | 0.0000 | Y |
| PDf | 15 | CPRD„ 201714 | England | 7 | -27 | 71 | 126 | 77 | 0.9880 | 0.0001 | Y |
| PDm | 16 | CPRD„ 201714 | England | 8 | -29 | 72 | 241 | 77 | 0.9996 | 0.0000 | Y |
| PD | 17 | Gov. of Alberta, 201838 | Alberta-2000 | 5 | -17 | 72 | 185 | 77 | 0.9223 | 0.0000 | Y |
| PD | 18 | Gov. of Alberta, 201838 | Alberta-2001 | 5 | -18 | 71 | 140 | 77 | 0.9267 | 0.0000 | Y |
| PD | 19 | Gov. of Alberta, 201838 | Alberta-2002 | 4 | -15 | 71 | 159 | 77 | 0.8982 | 0.0001 | Y |
| PD | 20 | Gov. of Alberta, 201838 | Alberta-2003 | 5 | -16 | 71 | 165 | 77 | 0.9250 | 0.0000 | Y |
| PD | 21 | Gov. of Alberta, 201838 | Alberta-2004 | 4 | -15 | 71 | 144 | 77 | 0.8687 | 0.0003 | Y |
| PD | 22 | Gov. of Alberta, 201838 | Alberta-2005 | 5 | -19 | 71 | 187 | 77 | 0.9739 | 0.0000 | Y |
| PD | 23 | Gov. of Alberta, 201838 | Alberta-2006 | 5 | -17 | 71 | 170 | 77 | 0.8622 | 0.0003 | Y |
| PD | 24 | Gov. of Alberta, 201838 | Alberta-2007 | 6 | -20 | 71 | 163 | 77 | 0.9369 | 0.0000 | Y |
| PD | 25 | Gov. of Alberta, 201838 | Alberta-2008 | 4 | -14 | 71 | 153 | 77 | 0.8588 | 0.0003 | Y |
| PD | 26 | Gov. of Alberta, 201838 | Alberta-2009 | 5 | -15 | 71 | 169 | 77 | 0.9161 | 0.0001 | Y |
| PD | 27 | Gov. of Alberta, 201838 | Alberta-2010 | 5 | -17 | 71 | 183 | 77 | 0.9298 | 0.0000 | Y |
| PD | 28 | Gov. of Alberta, 201838 | Alberta-2011 | 5 | -17 | 71 | 197 | 77 | 0.8867 | 0.0001 | Y |
| PD | 29 | Gov. of Alberta, 201838 | Alberta-2012 | 5 | -15 | 71 | 188 | 77 | 0.9005 | 0.0001 | Y |
| PD | 30 | Gov. of Alberta, 201838 | Alberta-2013 | 5 | -16 | 70 | 146 | 77 | 0.9386 | 0.0000 | Y |
| PD | 31 | Gov. of Alberta, 201838 | Alberta-2014 | 4 | -15 | 71 | 164 | 77 | 0.8512 | 0.0004 | Y |
| PD | 32 | Gov. of Alberta, 201838 | Alberta-2015 | 5 | -17 | 71 | 185 | 77 | 0.8944 | 0.0001 | Y |
| PD | 33 | Gov. of Alberta, 201838 | Alberta-2016 | 5 | -18 | 72 | 226 | 77 | 0.9428 | 0.0000 | Y |
| PD | 34 | Gov. of Alberta, 201838 | Alberta-2017 | 5 | -16 | 72 | 250 | 77 | 0.8698 | 0.0002 | Y |
| PDf | 35 | Gov. of Alberta, 201838 | Alberta-2000 | 5 | -18 | 72 | 140 | 77 | 0.9341 | 0.0000 | Y |
| PDf | 36 | Gov. of Alberta, 201838 | Alberta-2001 | 5 | -18 | 71 | 108 | 77 | 0.9154 | 0.0001 | Y |
| PDf | 37 | Gov. of Alberta, 201838 | Alberta-2002 | 5 | -18 | 71 | 110 | 77 | 0.8877 | 0.0001 | Y |
| PDf | 38 | Gov. of Alberta, 201838 | Alberta-2003 | 4 | -14 | 71 | 147 | 77 | 0.8866 | 0.0001 | Y |
| PDf | 39 | Gov. of Alberta, 201838 | Alberta-2004 | 4 | -13 | 70 | 103 | 77 | 0.8217 | 0.0008 | Y |
| PDf | 40 | Gov. of Alberta, 201838 | Alberta-2005 | 8 | -28 | 70 | 124 | 77 | 0.9378 | 0.0001 | Y |
| PDf | 41 | Gov. of Alberta, 201838 | Alberta-2006 | 5 | -17 | 71 | 137 | 77 | 0.8050 | 0.0010 | Y |
| PDf | 42 | Gov. of Alberta, 201838 | Alberta-2007 | 5 | -19 | 71 | 109 | 77 | 0.9399 | 0.0000 | Y |
| PDf | 43 | Gov. of Alberta, 201838 | Alberta-2008 | 4 | -14 | 71 | 125 | 77 | 0.8581 | 0.0003 | Y |
| PDf | 44 | Gov. of Alberta, 201838 | Alberta-2009 | 4 | -14 | 71 | 130 | 77 | 0.8458 | 0.0004 | Y |
| PDf | 45 | Gov. of Alberta, 201838 | Alberta-2010 | 5 | -19 | 71 | 122 | 77 | 0.9578 | 0.0000 | Y |
| PDf | 46 | Gov. of Alberta, 201838 | Alberta-2011 | 5 | -17 | 71 | 148 | 77 | 0.8464 | 0.0004 | Y |
| PDf | 47 | Gov. of Alberta, 201838 | Alberta-2012 | 4 | -13 | 71 | 124 | 77 | 0.8454 | 0.0005 | Y |
| PDf | 48 | Gov. of Alberta, 201838 | Alberta-2013 | 5 | -16 | 71 | 107 | 77 | 0.9346 | 0.0000 | Y |
| PDf | 49 | Gov. of Alberta, 201838 | Alberta-2014 | 5 | -15 | 70 | 107 | 77 | 0.9172 | 0.0000 | Y |
| PDf | 50 | Gov. of Alberta, 201838 | Alberta-2015 | 5 | -16 | 71 | 113 | 77 | 0.8439 | 0.0005 | Y |
| PDf | 51 | Gov. of Alberta, 201838 | Alberta-2016 | 5 | -17 | 71 | 166 | 77 | 0.8553 | 0.0004 | Y |
| PDf | 52 | Gov. of Alberta, 201838 | Alberta-2017 | 5 | -17 | 71 | 147 | 77 | 0.8974 | 0.0001 | Y |
| PDm | 53 | Gov. of Alberta, 201838 | Alberta-2000 | 5 | -16 | 72 | 245 | 77 | 0.9028 | 0.0001 | Y |
| PDm | 54 | Gov. of Alberta, 201838 | Alberta-2001 | 5 | -18 | 70 | 181 | 77 | 0.9185 | 0.0000 | Y |
| PDm | 55 | Gov. of Alberta, 201838 | Alberta-2002 | 4 | -14 | 71 | 223 | 77 | 0.8759 | 0.0002 | Y |
| PDm | 56 | Gov. of Alberta, 201838 | Alberta-2003 | 6 | -20 | 71 | 189 | 77 | 0.9048 | 0.0001 | Y |
| PDm | 57 | Gov. of Alberta, 201838 | Alberta-2004 | 5 | -17 | 71 | 195 | 77 | 0.8913 | 0.0001 | Y |
| PDm | 58 | Gov. of Alberta, 201838 | Alberta-2005 | 5 | -18 | 71 | 263 | 77 | 0.9600 | 0.0000 | Y |
| PDm | 59 | Gov. of Alberta, 201838 | Alberta-2006 | 5 | -17 | 71 | 210 | 77 | 0.8934 | 0.0001 | Y |
| PDm | 60 | Gov. of Alberta, 201838 | Alberta-2007 | 6 | -21 | 71 | 226 | 77 | 0.9144 | 0.0001 | Y |
| PDm | 61 | Gov. of Alberta, 201838 | Alberta-2008 | 4 | -15 | 70 | 185 | 77 | 0.7876 | 0.0014 | Y |
| PDm | 62 | Gov. of Alberta, 201838 | Alberta-2009 | 5 | -17 | 71 | 215 | 77 | 0.9495 | 0.0000 | Y |
| PDm | 63 | Gov. of Alberta, 201838 | Alberta-2010 | 5 | -17 | 71 | 255 | 77 | 0.9062 | 0.0001 | Y |
| PDm | 64 | Gov. of Alberta, 201838 | Alberta-2011 | 5 | -18 | 71 | 256 | 77 | 0.8860 | 0.0002 | Y |
| PDm | 65 | Gov. of Alberta, 201838 | Alberta-2012 | 5 | -18 | 72 | 262 | 77 | 0.9437 | 0.0000 | Y |
| PDm | 66 | Gov. of Alberta, 201838 | Alberta-2013 | 5 | -15 | 71 | 192 | 77 | 0.9166 | 0.0001 | Y |
| PDm | 67 | Gov. of Alberta, 201838 | Alberta-2014 | 4 | -15 | 71 | 233 | 77 | 0.7827 | 0.0015 | Y |
| PDm | 68 | Gov. of Alberta, 201838 | Alberta-2015 | 5 | -18 | 72 | 271 | 77 | 0.8966 | 0.0001 | Y |
| PDm | 69 | Gov. of Alberta, 201838 | Alberta-2016 | 6 | -21 | 72 | 299 | 77 | 0.9738 | 0.0000 | Y |
| PDm | 70 | Gov. of Alberta, 201838 | Alberta-2017 | 5 | -16 | 73 | 374 | 77 | 0.8379 | 0.0005 | Y |
| PD | 71 | Horsfall *et al*. 201347 | UK | 7 | -24 | 78 | 233 | 85 | 0.9671 | 0.0166 | Y |
| PD | 72 | Horsfall *et al*. 201347 | UK | 6 | -20 | 78 | 339 | 85 | 0.9901 | 0.0049 | Y |
| PD | 73 | Driver *et al*. 200925 | US | 8 | -28 | 70 | 255 | 77 | 0.9832 | 0.0000 | Y |
| PDm | 74 | Tan *et al*. 200787 | Singapore | 3 | -7 | 67 | 51 | 65 | 0.7113 | 0.3611 | N |
| PDf | 75 | Tan *et al*. 200787 | Singapore | 1 | 2 | 70 | 62 | 75 | 1.0000 | NaN | Y |
| PD | 76 | Tan *et al*. 200787 | China | 5 | -18 | 76 | 19 | 75 | 0.7566 | 0.1302 | N |
| PD | 77 | Tan *et al*. 200787 | Ferrara, Italy | 0 | 3 | 69 | 36 | 65 | 0.0089 | 0.9054 | N |
| PD | 78 | Tan *et al*. 200787 | Navarra, Spain | 4 | -12 | 74 | 42 | 75 | 0.7049 | 0.1604 | N |
| PD | 79 | Tan *et al*. 200787 | Sweden | 5 | -19 | 77 | 79 | 85 | 0.9760 | 0.0121 | Y |
| PD | 80 | Tan *et al*. 200787 | Singapore | 6 | -20 | 68 | 56 | 75 | 0.8025 | 0.2932 | N |
| PD | 81 | Tan *et al*. 200787 | Ilan county, Taiwan | 6 | -19 | 69 | 100 | 75 | 0.9994 | 0.0155 | Y |
| PD | 82 | Tan *et al*. 200787 | Poznan, Poland | 1 | 0 | 70 | 75 | 75 | 0.0984 | 0.6863 | N |
| PDm | 83 | Tan *et al*. 200787 | Hawaii, US, Japanese men | 4 | -12 | 75 | 107 | 85 | 0.9475 | 0.0266 | Y |
| PD | 84 | Tan *et al*. 200787 | Olmstead County, US | 4 | -12 | 74 | 93 | 75 | 0.8174 | 0.0959 | N |
| PD | 85 | Tan *et al*. 200787 | London, UK | 4 | -12 | 77 | 161 | 75 | 0.5595 | 0.4620 | N |
| PD | 86 | Tan *et al*. 200787 | Manhatten, US | 7 | -25 | 78 | 213 | 85 | 0.9654 | 0.0175 | Y |
| PD | 87 | de Lau *et al*. 2004101 | General | 7 | -24 | 82 | 430 | 90 | 0.9379 | 0.0316 | Y |
| PDm | 88 | de Lau *et al*. 2004101 | General | 8 | -28 | 84 | 950 | 90 | 0.9534 | 0.0236 | Y |
| PDf | 89 | de Lau *et al*. 2004101 | General | 6 | -21 | 80 | 260 | 90 | 0.7669 | 0.1243 | N |
| PD | 90 | Perez *et al*. 201077 | Gironde-Dordogne-France | 0 | 7 | 74 | 345 | 67 | 0.0090 | 0.9049 | N |
| PDm | 91 | Das *et al*. 201022 | Kolkata-India | 4 | -12 | 63 | 44 | 72 | 0.8700 | 0.0022 | Y |
| PDf | 92 | Das *et al*. 201022 | Kolkata-India | 4 | -11 | 69 | 93 | 77 | 0.9194 | 0.0100 | Y |
| PD | 93 | Das *et al*. 201022 | Kolkata-India | 3 | -10 | 67 | 47 | 77 | 0.6539 | 0.0151 | Y |
| PDm | 94 | Duncan *et al*. 201426 | Newcastle-Gateshead | 8 | -30 | 71 | 156 | 75 | 0.9765 | 0.0015 | Y |
| PDf | 95 | Duncan *et al*. 201426 | Newcastle-Gateshead | 6 | -23 | 69 | 72 | 75 | 0.9434 | 0.0058 | Y |
| PD | 96 | Duncan *et al*. 201426 | Newcastle-Gateshead | 7 | -26 | 71 | 110 | 75 | 0.9825 | 0.0010 | Y |
| PD | 97 | Liu *et al*. 201657 | Taiwan, 2005 | 7 | -24 | 77 | 402 | 85 | 0.9298 | 0.0357 | Y |
| PD | 98 | Liu *et al*. 201657 | Taiwan, 2006 | 7 | -24 | 77 | 386 | 85 | 0.9392 | 0.0309 | Y |
| PD | 99 | Liu *et al*. 201657 | Taiwan-2007 | 7 | -24 | 77 | 382 | 85 | 0.9422 | 0.0293 | Y |
| PD | 100 | Liu *et al*. 201657 | Taiwan, 2008 | 7 | -23 | 77 | 344 | 85 | 0.9371 | 0.0320 | Y |
| PD | 101 | Liu *et al*. 201657 | Taiwan, 2009 | 7 | -24 | 77 | 346 | 85 | 0.9402 | 0.0304 | Y |
| PD | 102 | Liu *et al*. 201657 | Taiwan, 2010 | 7 | -24 | 78 | 365 | 85 | 0.9531 | 0.0237 | Y |
| PD | 103 | Liu *et al*. 201657 | Taiwan, 2011 | 7 | -25 | 78 | 364 | 85 | 0.9585 | 0.0210 | Y |
| PD | 104 | Alves *et al*. 20092 | Norway | 7 | -24 | 68 | 83 | 75 | 0.9682 | 0.0160 | Y |
| PDm | 105 | Alves *et al*. 20092 | Norway | 7 | -24 | 68 | 100 | 75 | 0.9651 | 0.0176 | Y |
| PDf | 106 | Alves *et al*. 20092 | Norway | 7 | -25 | 69 | 68 | 75 | 0.9766 | 0.0118 | Y |
| PD | 107 | Gordon *et al*. 201536 | Navajo Nation | 5 | -19 | 76 | 232 | 80 | 0.9975 | 0.0321 | Y |
| PDm | 108 | Pupillo *et al*. 201678 | Italy | 4 | -15 | 75 | 143 | 80 | 0.8489 | 0.0090 | Y |
| PDf | 109 | Pupillo *et al*. 201678 | Italy | 7 | -25 | 75 | 102 | 80 | 0.9683 | 0.0024 | Y |
| PD | 110 | Pupillo *et al*. 201678 | Italy | 5 | -18 | 75 | 119 | 80 | 0.9149 | 0.0028 | Y |
| PD | 111 | Evans *et al*. 201629 | Cambridgeshire, UK-CamPalGN | 6 | -23 | 69 | 76 | 75 | 0.9722 | 0.0020 | Y |
| PD | 112 | Evans *et al*. 201629 | Cambridgeshire, UK-PICNICS | 7 | -27 | 69 | 80 | 75 | 0.9681 | 0.0161 | Y |
| PD | 113 | Fleury *et al*. 201831 | Geneva, Switzerland | 6 | -20 | 69 | 85 | 75 | 0.9924 | 0.0038 | Y |
| PDm | 114 | Fleury *et al*. 201831 | Geneva, Switzerland | 5 | -19 | 69 | 112 | 75 | 0.9925 | 0.0037 | Y |
| PDf | 115 | Fleury *et al*. 201831 | Geneva, Switzerland | 6 | -23 | 70 | 63 | 75 | 0.9846 | 0.0077 | Y |
| PDMf | 1 | de Lau *et al*. 2004101 | General | 8 | -30 | 83 | 1020 | 90 | 0.8988 | 0.0519 | N |
| PDMm | 2 | de Lau *et al*. 2004101 | General | 7 | -27 | 84 | 1210 | 90 | 0.9639 | 0.0182 | Y |
| PDM | 3 | de Lau *et al*. 2004101 | General | 7 | -26 | 83 | 1060 | 90 | 0.9783 | 0.0109 | Y |
| PDM | 4 | Perez *et al*. 201077 | Gironde-Dordogne-France | 4 | -10 | 76 | 762 | 82 | 0.6135 | 0.2168 | N |
| PDD | 1 | Savica *et al*. 201384 | Olmsted | 10 | -42 | 80 | 47 | 85 | 0.9914 | 0.0043 | Y |
| PDDm | 2 | Savica *et al*. 201384 | Olmsted | 10 | -40 | 81 | 68 | 85 | 0.9188 | 0.0415 | Y |
| PDDf | 3 | Savica *et al*. 201384 | Olmsted | 8 | -30 | 80 | 38 | 85 | 0.9743 | 0.1025 | N |
| MSm | 1 | Mackenzie *et al*. 201465 | UK | 1 | -4 | 50 | 10 | 45 | 0.6805 | 0.0224 | Y |
| MSf | 2 | Mackenzie *et al*. 201465 | UK | 1 | -2 | 46 | 26 | 45 | 0.4077 | 0.1227 | N |
| MSm | 3 | Barnett *et al*. 20037 | Newcastle, Australia-1971-1981 | 1 | -2 | 32 | 5 | 25 | 0.4627 | 0.3198 | N |
| MSf | 4 | Barnett *et al*. 20037 | Newcastle, Australia-1971-1981 | 1 | -1 | 31 | 6 | 35 | 0.1747 | 0.5820 | N |
| MS | 5 | Barnett *et al*. 20037 | Newcastle, Australia-1971-1981 | 1 | -1 | 31 | 5 | 25 | 0.4770 | 0.3094 | N |
| MSm | 6 | Barnett *et al*. 20037 | Newcastle, Australia-1986-1996 | -5 | 16 | 36 | 6 | 35 | 0.1086 | 0.6704 | N |
| MSf | 7 | Barnett *et al*. 20037 | Newcastle, Australia-1986-1996 | -1 | 4 | 42 | 6 | 55 | 0.3717 | 0.2749 | N |
| MS | 8 | Barnett *et al*. 20037 | Newcastle, Australia-1986-1996 | -1 | 4 | 42 | 6 | 35 | 0.3335 | 0.3079 | N |
| MSf | 9 | Hernán *et al*. 199946 | USA | -1 | 7 | 43 | 19 | 42 | 0.5238 | 0.0275 | Y |
| MSf | 10 | Hernán *et al*. 199946 | USA | -3 | 12 | 39 | 18 | 27 | 0.8540 | 0.0004 | Y |
| MSm | 11 | Nicoletti *et al*. 201171 | Catania | 4 | -19 | 86 | 3 | 92 | 0.8763 | 0.0639 | N |
| MSf | 12 | Nicoletti *et al*. 201171 | Catania | 7 | -30 | 87 | 5 | 92 | 0.9589 | 0.0208 | Y |
| MS | 13 | Nicoletti *et al*. 201171 | Catania | 6 | -26 | 87 | 4 | 92 | 0.9667 | 0.0168 | Y |
| MSm | 14 | Nicoletti *et al*. 201171 | Catania | 0 | 1 | 40 | 11 | 50 | 0.0958 | 0.6124 | N |
| MSf | 15 | Nicoletti *et al*. 201171 | Catania | -2 | 9 | 34 | 24 | 30 | 0.4244 | 0.2337 | N |
| MS | 16 | Nicoletti *et al*. 201171 | Catania | -1 | 5 | 36 | 16 | 30 | 0.1918 | 0.4607 | N |
| MSm | 17 | Nicoletti *et al*. 200172 | Catania | 1 | -2 | 31 | 5 | 30 | 0.1028 | 0.4831 | N |
| MSf | 18 | Nicoletti *et al*. 200172 | Catania | 3 | -11 | 31 | 7 | 30 | 0.3728 | 0.1453 | N |
| MS | 19 | Nicoletti *et al*. 200172 | Catania | 1 | -2 | 31 | 6 | 30 | 0.1310 | 0.4250 | N |
| MSm | 20 | Sarasoja *et al*. 200483 | Central Finland | 1 | -4 | 41 | 7 | 45 | 0.3733 | 0.1976 | N |
| MSf | 21 | Sarasoja *et al*. 200483 | Central Finland | 1 | -2 | 39 | 14 | 35 | 0.1713 | 0.4147 | N |
| MS | 22 | Sarasoja *et al*. 200483 | Central Finland | 1 | -3 | 40 | 10 | 45 | 0.2276 | 0.3387 | N |
| MSm | 23 | Svenningsson *et al*. 199086 | Gothenburg-1950-64 | -1 | 4 | 33 | 8 | 25 | 0.1970 | 0.3780 | N |
| MSf | 24 | Svenningsson *et al*. 199086 | Gothenburg-1950-64 | -4 | 16 | 33 | 13 | 35 | 0.2854 | 0.2749 | N |
| MSm | 25 | Svenningsson *et al*. 199086 | Gothenburg-1974-88 | 0 | 1 | 36 | 7 | 35 | 0.0038 | 0.9079 | N |
| MSf | 26 | Svenningsson *et al*. 199086 | Gothenburg-1974-88 | -1 | 3 | 34 | 8 | 35 | 0.1946 | 0.3812 | N |
| MSm | 27 | Svenningsson *et al*. 199086 | Gothenburg-1974-78 | -4 | 14 | 34 | 6 | 35 | 0.2918 | 0.2685 | N |
| MSf | 28 | Svenningsson *et al*. 199086 | Gothenburg-1974-78 | -1 | 4 | 34 | 10 | 25 | 0.2239 | 0.3431 | N |
| MSm | 29 | Svenningsson *et al*. 199086 | Gothenburg-1979-83 | 5 | -19 | 37 | 10 | 35 | 0.4474 | 0.1463 | N |
| MSf | 30 | Svenningsson *et al*. 199086 | Gothenburg-1979-83 | -1 | 5 | 32 | 8 | 25 | 0.4341 | 0.1547 | N |
| MSm | 31 | Svenningsson *et al*. 199086 | Gothenburg-1984-88 | 2 | -9 | 37 | 6 | 35 | 0.0325 | 0.7325 | N |
| MSf | 32 | Svenningsson *et al*. 199086 | Gothenburg-1984-88 | 5 | -20 | 38 | 7 | 35 | 0.4703 | 0.1326 | N |
| MS | 33 | Gov. of Alberta, 201837 | Alberta-2000 | 0 | 2 | 46 | 46 | 42 | 0.0073 | 0.8143 | N |
| MS | 34 | Gov. of Alberta, 201837 | Alberta-2001 | 0 | 3 | 46 | 41 | 52 | 0.0004 | 0.9551 | N |
| MS | 35 | Gov. of Alberta, 201837 | Alberta-2002 | 0 | 3 | 46 | 34 | 42 | 0.0011 | 0.9276 | N |
| MS | 36 | Gov. of Alberta, 201837 | Alberta-2003 | 0 | 3 | 46 | 36 | 42 | 0.0000 | 0.9867 | N |
| MS | 37 | Gov. of Alberta, 201837 | Alberta-2004 | 0 | 3 | 47 | 37 | 37 | 0.0002 | 0.9681 | N |
| MS | 38 | Gov. of Alberta, 201837 | Alberta-2005 | 0 | 2 | 49 | 32 | 47 | 0.0373 | 0.5694 | N |
| MS | 39 | Gov. of Alberta, 201837 | Alberta-2006 | 0 | 3 | 48 | 31 | 42 | 0.0000 | 0.9912 | N |
| MS | 40 | Gov. of Alberta, 201837 | Alberta-2007 | 0 | 3 | 48 | 34 | 47 | 0.0018 | 0.9022 | N |
| MS | 41 | Gov. of Alberta, 201837 | Alberta-2008 | 0 | 3 | 48 | 33 | 47 | 0.0028 | 0.8764 | N |
| MS | 42 | Gov. of Alberta, 201837 | Alberta-2009 | -1 | 5 | 46 | 27 | 42 | 0.0810 | 0.3963 | N |
| MS | 43 | Gov. of Alberta, 201837 | Alberta-2010 | 0 | 2 | 49 | 26 | 47 | 0.0072 | 0.8036 | N |
| MS | 44 | Gov. of Alberta, 201837 | Alberta-2011 | 0 | 3 | 47 | 28 | 47 | 0.0091 | 0.7804 | N |
| MS | 45 | Gov. of Alberta, 201837 | Alberta-2012 | 0 | 3 | 47 | 29 | 32 | 0.0174 | 0.6994 | N |
| MS | 46 | Gov. of Alberta, 201837 | Alberta-2013 | 0 | 4 | 47 | 27 | 52 | 0.0269 | 0.6296 | N |
| MS | 47 | Gov. of Alberta, 201837 | Alberta-2014 | 0 | 2 | 46 | 30 | 37 | 0.0071 | 0.8170 | N |
| MS | 48 | Gov. of Alberta, 201837 | Alberta-2015 | 0 | 3 | 47 | 29 | 47 | 0.0187 | 0.6883 | N |
| MS | 49 | Gov. of Alberta, 201837 | Alberta-2016 | 0 | 3 | 48 | 29 | 47 | 0.0129 | 0.7399 | N |
| MS | 50 | Gov. of Alberta, 201837 | Alberta-2017 | 0 | 3 | 48 | 28 | 42 | 0.0006 | 0.9445 | N |
| MSf | 51 | Gov. of Alberta, 201837 | Alberta-2000 | 0 | 3 | 46 | 66 | 42 | 0.0085 | 0.8001 | N |
| MSf | 52 | Gov. of Alberta, 201837 | Alberta-2001 | 0 | 4 | 45 | 60 | 52 | 0.0066 | 0.8229 | N |
| MSf | 53 | Gov. of Alberta, 201837 | Alberta-2002 | 0 | 4 | 45 | 53 | 42 | 0.0237 | 0.6712 | N |
| MSf | 54 | Gov. of Alberta, 201837 | Alberta-2003 | 0 | 4 | 44 | 61 | 42 | 0.0312 | 0.6252 | N |
| MSf | 55 | Gov. of Alberta, 201837 | Alberta-2004 | 0 | 4 | 46 | 50 | 52 | 0.0246 | 0.6450 | N |
| MSf | 56 | Gov. of Alberta, 201837 | Alberta-2005 | 0 | 3 | 47 | 52 | 47 | 0.0006 | 0.9415 | N |
| MSf | 57 | Gov. of Alberta, 201837 | Alberta-2006 | 0 | 4 | 47 | 50 | 42 | 0.0087 | 0.7855 | N |
| MSf | 58 | Gov. of Alberta, 201837 | Alberta-2007 | -1 | 5 | 46 | 51 | 47 | 0.0713 | 0.4274 | N |
| MSf | 59 | Gov. of Alberta, 201837 | Alberta-2008 | 0 | 4 | 47 | 51 | 47 | 0.0238 | 0.6504 | N |
| MSf | 60 | Gov. of Alberta, 201837 | Alberta-2009 | 0 | 5 | 46 | 42 | 37 | 0.0557 | 0.4849 | N |
| MSf | 61 | Gov. of Alberta, 201837 | Alberta-2010 | 0 | 3 | 48 | 43 | 42 | 0.0006 | 0.9439 | N |
| MSf | 62 | Gov. of Alberta, 201837 | Alberta-2011 | 0 | 4 | 46 | 40 | 42 | 0.0421 | 0.5449 | N |
| MSf | 63 | Gov. of Alberta, 201837 | Alberta-2012 | 0 | 4 | 46 | 43 | 32 | 0.0428 | 0.5415 | N |
| MSf | 64 | Gov. of Alberta, 201837 | Alberta-2013 | 0 | 4 | 47 | 40 | 37 | 0.0380 | 0.5659 | N |
| MSf | 65 | Gov. of Alberta, 201837 | Alberta-2014 | 0 | 3 | 46 | 45 | 47 | 0.0002 | 0.9680 | N |
| MSf | 66 | Gov. of Alberta, 201837 | Alberta-2015 | 0 | 5 | 47 | 43 | 52 | 0.0434 | 0.5390 | N |
| MSf | 67 | Gov. of Alberta, 201837 | Alberta-2016 | 0 | 4 | 47 | 41 | 42 | 0.0403 | 0.5538 | N |
| MSf | 68 | Gov. of Alberta, 201837 | Alberta-2017 | 0 | 4 | 48 | 40 | 37 | 0.0204 | 0.6756 | N |
| MSm | 69 | Gov. of Alberta, 201837 | Alberta-2000 | 0 | 1 | 45 | 26 | 42 | 0.0444 | 0.5863 | N |
| MSm | 70 | Gov. of Alberta, 201837 | Alberta-2001 | 0 | 1 | 49 | 26 | 47 | 0.0308 | 0.6279 | N |
| MSm | 71 | Gov. of Alberta, 201837 | Alberta-2002 | 0 | 1 | 48 | 19 | 47 | 0.0860 | 0.4110 | N |
| MSm | 72 | Gov. of Alberta, 201837 | Alberta-2003 | 1 | 0 | 49 | 17 | 57 | 0.1883 | 0.2102 | N |
| MSm | 73 | Gov. of Alberta, 201837 | Alberta-2004 | 0 | 1 | 50 | 25 | 37 | 0.0938 | 0.3598 | N |
| MSm | 74 | Gov. of Alberta, 201837 | Alberta-2005 | 1 | -1 | 53 | 15 | 42 | 0.4824 | 0.0177 | Y |
| MSm | 75 | Gov. of Alberta, 201837 | Alberta-2006 | 0 | 1 | 50 | 18 | 52 | 0.0756 | 0.4131 | N |
| MSm | 76 | Gov. of Alberta, 201837 | Alberta-2007 | 1 | 0 | 51 | 24 | 52 | 0.1274 | 0.2813 | N |
| MSm | 77 | Gov. of Alberta, 201837 | Alberta-2008 | 0 | 2 | 49 | 21 | 37 | 0.0063 | 0.8164 | N |
| MSm | 78 | Gov. of Alberta, 201837 | Alberta-2009 | 0 | 2 | 45 | 16 | 42 | 0.0047 | 0.8610 | N |
| MSm | 79 | Gov. of Alberta, 201837 | Alberta-2010 | 0 | 1 | 50 | 15 | 47 | 0.0843 | 0.3863 | N |
| MSm | 80 | Gov. of Alberta, 201837 | Alberta-2011 | 0 | 1 | 49 | 19 | 47 | 0.0131 | 0.7379 | N |
| MSm | 81 | Gov. of Alberta, 201837 | Alberta-2012 | 1 | 0 | 49 | 18 | 47 | 0.2000 | 0.1950 | N |
| MSm | 82 | Gov. of Alberta, 201837 | Alberta-2013 | 0 | 1 | 48 | 19 | 42 | 0.1137 | 0.3408 | N |
| MSm | 83 | Gov. of Alberta, 201837 | Alberta-2014 | 0 | 1 | 46 | 20 | 42 | 0.0630 | 0.4844 | N |
| MSm | 84 | Gov. of Alberta, 201837 | Alberta-2015 | 0 | 2 | 49 | 17 | 37 | 0.0001 | 0.9766 | N |
| MSm | 85 | Gov. of Alberta, 201837 | Alberta-2016 | 1 | 0 | 50 | 20 | 47 | 0.4223 | 0.0419 | Y |
| MSm | 86 | Gov. of Alberta, 201837 | Alberta-2017 | 0 | 1 | 49 | 21 | 47 | 0.0374 | 0.5691 | N |
| MSm | 87 | O’Connell *et al*. 201775 | Ireland | -1 | 6 | 43 | 7 | 35 | 0.5617 | 0.0863 | N |
| MSf | 88 | O’Connell *et al*. 201775 | Ireland | 1 | -2 | 40 | 21 | 35 | 0.2868 | 0.2735 | N |
| MS | 89 | O’Connell *et al*. 201775 | Ireland | 0 | 0 | 40 | 14 | 35 | 0.0108 | 0.8245 | N |
| MSm | 90 | Caniglia-Tenaglia *et al*. 201816 | San Marino-2005-2014 | 1 | -3 | 38 | 15 | 45 | 1.0000 | NaN | Y |
| MSf | 91 | Caniglia-Tenaglia *et al*. 201816 | San Marino-2005-2014 | 0 | 3 | 33 | 33 | 25 | 0.0181 | 0.8291 | N |
| MS | 92 | Caniglia-Tenaglia *et al*. 201816 | San Marino-2005-2014 | 0 | 2 | 34 | 20 | 25 | 0.0665 | 0.6754 | N |
| MSm | 93 | Palese *et al*. 201876 | Friuli-Venezia Giulia, Italy-2005-2014 | 3 | -10 | 69 | 10 | 70 | 0.9760 | 0.0016 | Y |
| MSf | 94 | Palese *et al*. 201876 | Friuli-Venezia Giulia, Italy-2005-2014 | 3 | -11 | 69 | 8 | 70 | 0.8930 | 0.0154 | Y |
| MS | 95 | Palese *et al*. 201876 | Friuli-Venezia Giulia, Italy-2005-2014 | 3 | -10 | 69 | 9 | 70 | 0.9523 | 0.0045 | Y |
| MS | 96 | Simonsen *et al*. 201785 | Norway-Buskerud | 1 | 0 | 50 | 25 | 55 | 0.0719 | 0.6074 | N |
| AD | 1 | Andreasen *et al*. 19994 | Piteå River Valley, Sweden-1990-96 | 9 | -35 | 74 | 481 | 77 | 0.9845 | 0.0078 | Y |
| AD | 2 | Hebert *et al*. 199542 | East Boston, Mass., USA | 9 | -30 | 78 | 3300 | 82 | 0.9951 | 0.0025 | Y |
| AD | 3 | Nilsson *et al*. 201473 | Sweden | 8 | -31 | 72 | 145 | 77 | 0.9802 | 0.0001 | Y |
| AD | 4 | Jorm & Jolley 199850 | Europe-Mild+ | 11 | -40 | 78 | 2210 | 82 | 0.9992 | 0.0004 | Y |
| AD | 5 | Jorm & Jolley 199850 | Europe-Moderate+ | 12 | -45 | 79 | 1060 | 82 | 0.9990 | 0.0005 | Y |
| AD | 6 | Jorm & Jolley 199850 | USA-Mild+ | 9 | -32 | 78 | 3840 | 82 | 0.9977 | 0.0012 | Y |
| AD | 7 | Jorm & Jolley 199850 | USA-Moderate+ | 11 | -42 | 78 | 1480 | 82 | 0.9992 | 0.0004 | Y |
| AD | 8 | Jorm & Jolley 199850 | East Asia-Mild+ | 15 | -60 | 79 | 1490 | 82 | 1.0000 | 0.0000 | Y |
| ADm | 9 | Rocca *et al*. 199880 | Rochester-1975 | 9 | -31 | 72 | 839 | 72 | 0.7460 | 0.1363 | N |
| ADm | 10 | Rocca *et al*. 199880 | Rochester-1976 | 4 | -13 | 75 | 556 | 77 | 1.0000 | NaN | Y |
| ADm | 11 | Rocca *et al*. 199880 | Rochester-1977 | 3 | -8 | 71 | 544 | 77 | 0.6911 | 0.1687 | N |
| ADm | 12 | Rocca *et al*. 199880 | Rochester-1978 | 15 | -57 | 76 | 535 | 77 | 1.0000 | NaN | Y |
| ADm | 13 | Rocca *et al*. 199880 | Rochester-1979 | 4 | -11 | 73 | 615 | 72 | 0.1602 | 0.7379 | N |
| ADm | 14 | Rocca *et al*. 199880 | Rochester-1975-79 | 10 | -36 | 73 | 495 | 72 | 0.9101 | 0.0460 | Y |
| ADm | 15 | Rocca *et al*. 199880 | Rochester-1980 | 4 | -12 | 73 | 402 | 72 | 0.3295 | 0.6108 | N |
| ADm | 16 | Rocca *et al*. 199880 | Rochester-1981 | 9 | -33 | 73 | 1026 | 77 | 0.8535 | 0.0762 | N |
| ADm | 17 | Rocca *et al*. 199880 | Rochester-1982 | 9 | -34 | 73 | 750 | 77 | 0.9518 | 0.0244 | Y |
| ADm | 18 | Rocca *et al*. 199880 | Rochester-1983 | 32 | -130 | 76 | 1478 | 77 | 1.0000 | NaN | Y |
| ADm | 19 | Rocca *et al*. 199880 | Rochester-1984 | 9 | -31 | 74 | 962 | 77 | 0.7037 | 0.1611 | N |
| ADm | 20 | Rocca *et al*. 199880 | Rochester-1980-84 | 12 | -44 | 74 | 904 | 77 | 0.9711 | 0.0145 | Y |
| ADm | 21 | Rocca *et al*. 199880 | Rochester-1975-84 | 11 | -41 | 73 | 704 | 77 | 0.9931 | 0.0035 | Y |
| ADf | 22 | Rocca *et al*. 199880 | Rochester-1975-79 | 19 | -76 | 75 | 833 | 77 | 0.9536 | 0.0235 | Y |
| ADf | 23 | Rocca *et al*. 199880 | Rochester-1980-84 | 12 | -44 | 74 | 804 | 77 | 0.9318 | 0.0077 | Y |
| ADf | 24 | Rocca *et al*. 199880 | Rochester-1975-84 | 14 | -54 | 75 | 818 | 77 | 0.9573 | 0.0038 | Y |
| ADf | 25 | Rocca *et al*. 199880 | Rochester-1975 | 21 | -86 | 76 | 1361 | 77 | 1.0000 | NaN | Y |
| ADf | 26 | Rocca *et al*. 199880 | Rochester-1976 | 8 | -29 | 75 | 932 | 77 | 1.0000 | NaN | Y |
| ADf | 27 | Rocca *et al*. 199880 | Rochester-1977 | 19 | -77 | 75 | 1430 | 77 | 0.9602 | 0.1278 | N |
| ADf | 28 | Rocca *et al*. 199880 | Rochester-1978 | 4 | -12 | 73 | 255 | 77 | 0.6485 | 0.4040 | N |
| ADf | 29 | Rocca *et al*. 199880 | Rochester-1979 | 7 | -24 | 73 | 305 | 72 | 0.6119 | 0.4281 | N |
| ADf | 30 | Rocca *et al*. 199880 | Rochester-1980 | 9 | -34 | 73 | 1340 | 77 | 0.9246 | 0.0090 | Y |
| ADf | 31 | Rocca *et al*. 199880 | Rochester-1981 | 17 | -66 | 75 | 959 | 77 | 0.9862 | 0.0749 | N |
| ADf | 32 | Rocca *et al*. 199880 | Rochester-1982 | -2 | 14 | 74 | 796 | 72 | 1.0000 | NaN | Y |
| ADf | 33 | Rocca *et al*. 199880 | Rochester-1983 | 8 | -28 | 75 | 582 | 77 | 0.6310 | 0.4156 | N |
| ADf | 34 | Rocca *et al*. 199880 | Rochester-1984 | 6 | -19 | 73 | 460 | 77 | 0.7801 | 0.3107 | N |
| AD | 35 | Perez *et al*. 201077 | Gironde-Dordogne, France | 17 | -67 | 79 | 2705 | 82 | 0.9816 | 0.0093 | Y |
| AD | 36 | Matsui *et al*. 200968 | Hisayama,Japan-1985–2002 | 9 | -29 | 78 | 4100 | 82 | 0.9715 | 0.0143 | Y |
| ADm | 37 | Bermejo-Pareja *et al*. 200811 | NEDICES-Central Spain | 9 | -33 | 77 | 840 | 82 | 0.7738 | 0.1203 | N |
| ADf | 38 | Bermejo-Pareja *et al*. 200811 | NEDICES-Central Spain | 14 | -55 | 80 | 2490 | 82 | 0.8613 | 0.0719 | N |
| AD | 39 | Bermejo-Pareja *et al*. 200811 | NEDICES-Central Spain | 12 | -47 | 79 | 1850 | 82 | 0.9928 | 0.0036 | Y |
| ADm | 40 | Chandra *et al*. 200118 | Ballabgarh, India | 10 | -39 | 87 | 2306 | 90 | 0.9371 | 0.1614 | N |
| ADf | 41 | Chandra *et al*. 200118 | Ballabgarh, India | 9 | -32 | 87 | 2925 | 90 | 0.8505 | 0.0778 | N |
| AD | 42 | Chandra *et al*. 200118 | Ballabgarh, India | 10 | -37 | 87 | 2481 | 90 | 0.9607 | 0.0198 | Y |
| AD | 43 | Chandra *et al*. 200118 | Monongahela Valley, USA | 8 | -28 | 86 | 5310 | 90 | 1.0000 | 0.0035 | Y |
| ADf | 44 | Ruitenberg *et al*. 200182 | Ommoord, Rotterdam, Netherlans-1990-1999 | 15 | -57 | 75 | 1060 | 77 | 0.9991 | 0.0187 | Y |
| ADm | 45 | Ruitenberg *et al*. 200182 | Ommoord, Rotterdam, Netherlans-1990-1999 | 16 | -63 | 75 | 860 | 77 | 0.9935 | 0.0033 | Y |
| AD | 46 | Ruitenberg *et al*. 200182 | Ommoord, Rotterdam, Netherlans-1990-1999 | 21 | -84 | 75 | 970 | 77 | 0.9479 | 0.0264 | Y |
| ADf | 47 | Kukull *et al*. 200254 | Siattle, Washington, USA | 14 | -52 | 80 | 3169 | 82 | 0.9442 | 0.0283 | Y |
| ADm | 48 | Kukull *et al*. 200254 | Siattle, Washington, USA | 9 | -30 | 78 | 2103 | 82 | 0.8396 | 0.0837 | N |
| ADm | 49 | Tyas *et al*. 200692 | Manitoba, Canada-MSHA | 4 | -10 | 78 | 960 | 82 | 0.8450 | 0.2576 | N |
| ADf | 50 | Tyas *et al*. 200692 | Manitoba, Canada-MSHA | 12 | -44 | 79 | 2740 | 82 | 0.6152 | 0.2156 | N |
| AD | 51 | Tyas *et al*. 200692 | Manitoba, Canada-MSHA | 12 | -45 | 79 | 2120 | 82 | 0.9723 | 0.0140 | Y |
| ADm | 52 | Di *et al*. 200223 | ILSA, Italy | 16 | -61 | 79 | 1562 | 82 | 0.9910 | 0.0045 | Y |
| ADf | 53 | Di *et al*. 200223 | ILSA, Italy | 8 | -29 | 77 | 1737 | 82 | 0.9595 | 0.0204 | Y |
| AD | 54 | Di *et al*. 200223 | ILSA, Italy | 11 | -39 | 78 | 1645 | 82 | 0.9926 | 0.0037 | Y |
| ADf | 55 | Edland *et al*. 200227 | Rochester, Minnesota, USA-1985-89 | 11 | -43 | 74 | 1009 | 77 | 0.9349 | 0.0016 | Y |
| ADm | 56 | Edland *et al*. 200227 | Rochester, Minnesota, USA-1985-89 | 10 | -37 | 74 | 948 | 77 | 0.8490 | 0.0090 | Y |
| AD | 57 | Edland *et al*. 200227 | Rochester, Minnesota, USA-1985-89 | 11 | -39 | 74 | 989 | 77 | 0.8914 | 0.0046 | Y |
| ADm | 58 | Ganguli *et al*. 200033 | MoVIES project-USA-CDR>=1.0 | 12 | -47 | 79 | 2630 | 82 | 0.9677 | 0.0163 | Y |
| ADf | 59 | Ganguli *et al*. 200033 | Monongahela,USA | 12 | -46 | 79 | 2550 | 82 | 0.9906 | 0.0047 | Y |
| AD | 60 | Ganguli *et al*. 200033 | MoVIES project-USA-CDR>=1.0 | 12 | -47 | 79 | 2580 | 82 | 0.9941 | 0.0029 | Y |
| AD | 61 | Hendrie *et al*. 200144 | Yoruba-Ibadan-Nigeria | 10 | -38 | 86 | 5020 | 90 | 0.9993 | 0.0171 | Y |
| AD | 62 | Hendrie *et al*. 200144 | African Americans-Indianapolis-USA | 7 | -20 | 84 | 7070 | 90 | 1.0000 | 0.0003 | Y |
| ADm | 63 | Kawas *et al*. 200052 | Baltimore, USA-BLSA | 15 | -60 | 74 | 750 | 77 | 0.8707 | 0.2342 | N |
| ADf | 64 | Kawas *et al*. 200052 | Baltimore, USA-BLSA | 6 | -18 | 73 | 1100 | 77 | 0.3847 | 0.3798 | N |
| AD | 65 | Kawas *et al*. 200052 | Baltimore-USA-BLSA | 12 | -44 | 74 | 890 | 77 | 0.9724 | 0.0139 | Y |
| ADf | 66 | Nitrini *et al*. 200474 | Catanduva, Sao Paulo, Brazil | 18 | -72 | 84 | 4100 | 87 | 0.9659 | 0.0172 | Y |
| ADm | 67 | Nitrini *et al*. 200474 | Catanduva, Sao Paulo, Brazil | 13 | -52 | 83 | 2147 | 82 | 0.8379 | 0.0846 | N |
| AD | 68 | Nitrini *et al*. 200474 | Catanduva, Sao Paulo, Brazil | 16 | -65 | 84 | 2965 | 87 | 0.9476 | 0.0266 | Y |
| ADm | 69 | López-Pousa *et al*. 200461 | Girona, Spain | 7 | -26 | 87 | 1720 | 92 | 0.9701 | 0.0151 | Y |
| ADf | 70 | López-Pousa *et al*. 200461 | Girona, Spain | 8 | -29 | 87 | 2750 | 92 | 0.9096 | 0.0463 | Y |
| AD | 71 | López-Pousa *et al*. 200461 | Girona, Spain | 8 | -29 | 87 | 2440 | 92 | 0.9632 | 0.0186 | Y |
| AD | 72 | Gao *et al*. 199834 | 12 studies | 14 | -54 | 74 | 1174 | 77 | 0.9984 | 0.0008 | Y |
| ADm | 73 | Imfeld *et al*. 201348 | UK | 10 | -40 | 78 | 232 | 82 | 0.9887 | 0.0057 | Y |
| ADf | 74 | Imfeld *et al*. 201348 | UK | 11 | -43 | 78 | 350 | 82 | 0.9965 | 0.0018 | Y |
| AD | 75 | Imfeld *et al*. 201348 | UK | 11 | -43 | 78 | 305 | 82 | 0.9951 | 0.0024 | Y |
| ADm | 76 | Ravaglia *et al*. 200579 | Conselice-Emilia Romagna-Italy | 6 | -20 | 85 | 5280 | 90 | 0.9413 | 0.1558 | N |
| ADf | 77 | Ravaglia *et al*. 200579 | Conselice-Emilia Romagna-Italy | 8 | -28 | 85 | 9680 | 90 | 1.0000 | 0.0007 | Y |
| AD | 78 | Ravaglia *et al*. 200579 | Conselice-Emilia Romagna-Italy | 8 | -25 | 85 | 7580 | 90 | 0.9963 | 0.0387 | Y |
| AD | 79 | Lobo *et al*. 201158 | Zaragoza, Spain | 14 | -56 | 75 | 430 | 77 | 0.9796 | 0.0912 | N |
| ADm | 80 | Lobo *et al*. 201158 | Zaragoza, Spain | 1 | 0 | 72 | 150 | 72 | NaN | NaN | Y |
| ADf | 81 | Lobo *et al*. 201158 | Zaragoza, Spain | 14 | -55 | 75 | 710 | 77 | 0.7698 | 0.3186 | N |
| AD | 82 | Andersen *et al*. 19993 | Odense, Denmark | 12 | -45 | 79 | 7410 | 82 | 0.9880 | 0.0060 | Y |
| ADm | 83 | Letenneur *et al*. 199956 | Gironde-Dordongne, France | 10 | -36 | 78 | 1330 | 82 | 0.8925 | 0.0553 | N |
| ADf | 84 | Letenneur *et al*. 199956 | Gironde-Dordongne, France | 16 | -63 | 80 | 2460 | 82 | 0.9921 | 0.0566 | N |
| ADm | 85 | Fratiglioni *et al*. 199732 | Kungsholmen Project, Stockholm, Sweden | 4 | -12 | 86 | 1500 | 92 | 0.6858 | 0.1719 | N |
| ADf | 86 | Fratiglioni *et al*. 199732 | Kungsholmen Project, Stockholm, Sweden | 11 | -41 | 88 | 7490 | 92 | 0.9148 | 0.0436 | Y |
| AD | 87 | Maestre *et al*. 201866 | Maracaibo, Venezuela | 10 | -37 | 86 | 6087 | 90 | 0.9654 | 0.0174 | Y |
| AD | 88 | Miech *et al*. 200269 | Cache-Utah-USA | 14 | -55 | 76 | 1884 | 79 | 0.9178 | 0.0103 | Y |
| ADm | 89 | Miech *et al*. 200269 | Cache-Utah-USA | 9 | -32 | 75 | 1063 | 76 | 0.7771 | 0.0481 | Y |
| ADf | 90 | Miech *et al*. 200269 | Cache-Utah-USA | 19 | -75 | 77 | 2424 | 79 | 0.9477 | 0.0052 | Y |
| HDf | 1 | Chen & Lai 201019 | Taiwan | 2 | -10 | 55 | 0 | 55 | 0.8424 | 0.0036 | Y |
| HDm | 2 | Chen & Lai 201019 | Taiwan | 2 | -8 | 52 | 0 | 45 | 0.6531 | 0.0279 | Y |
| HD | 3 | Chen & Lai 201019 | Taiwan | 2 | -9 | 53 | 0 | 45 | 0.8250 | 0.0047 | Y |
| HD | 4 | Wexler *et al*. 201697 | UK-1990-96 | 0 | -2 | 50 | 1 | 55 | 0.2097 | 0.5420 | N |
| HD | 5 | Wexler *et al*. 201697 | UK-1997-2003 | 1 | -4 | 52 | 1 | 55 | 0.5678 | 0.2465 | N |
| HD | 6 | Wexler *et al*. 201697 | UK-2004-2010 | 0 | -2 | 51 | 1 | 45 | 0.2517 | 0.4983 | N |
| HDm | 7 | Carrassi *et al*. 201717 | Ferrara-Italy-1990-2009 | 1 | -3 | 59 | 1 | 50 | 0.3624 | 0.3980 | N |
| HDf | 8 | Carrassi *et al*. 201717 | Ferrara-Italy-1990-2009 | 1 | -3 | 46 | 1 | 40 | 0.6921 | 0.1681 | N |
| CJD | 1 | Elsaadany *et al*. 200528 | Canada | 7 | -29 | 68 | 1 | 67 | 0.9416 | 0.0000 | Y |
| CJDm | 2 | Elsaadany *et al*. 200528 | Canada | 6 | -27 | 68 | 1 | 77 | 0.8822 | 0.0002 | Y |
| CJDf | 3 | Elsaadany *et al*. 200528 | Canada | 6 | -27 | 67 | 1 | 72 | 0.9104 | 0.0000 | Y |
| CJD | 4 | Begué *et al*. 20118 | Argentina | 4 | -19 | 64 | 0 | 65 | 0.6879 | 0.1706 | N |
| CJD | 5 | Heinemann *et al*. 200743 | Germany | 6 | -27 | 68 | 1 | 75 | 0.9425 | 0.0292 | Y |
| CJDm | 6 | Heinemann *et al*. 200743 | Germany | 6 | -27 | 67 | 1 | 75 | 0.9330 | 0.0341 | Y |
| CJDf | 7 | Heinemann *et al*. 200743 | Germany | 6 | -28 | 68 | 1 | 75 | 0.9614 | 0.0195 | Y |
| CJDm | 8 | Nakamura *et al*. 199970 | Japan | 5 | -22 | 66 | 0 | 75 | 0.9333 | 0.0075 | Y |
| CJDf | 9 | Nakamura *et al*. 199970 | Japan | 4 | -19 | 65 | 0 | 75 | 0.9613 | 0.0033 | Y |
| CJDm | 10 | Lu *et al*. 201060 | Taiwan | 4 | -19 | 67 | 0 | 65 | 0.9092 | 0.0119 | Y |
| CJDf | 11 | Lu *et al*. 201060 | Taiwan | 6 | -27 | 68 | 0 | 75 | 0.9634 | 0.0185 | Y |
| CJD | 12 | Lu *et al*. 201060 | Taiwan | 6 | -26 | 68 | 0 | 75 | 0.9628 | 0.0188 | Y |
| CJDm | 13 | Gubbels *et al*. 201240 | Sweden | 7 | -31 | 69 | 1 | 75 | 0.9682 | 0.0160 | Y |
| CJDf | 14 | Gubbels *et al*. 201240 | Sweden | 7 | -31 | 67 | 1 | 75 | 0.8963 | 0.0533 | N |
| CJD | 15 | Van *et al*. 201294 | Belgium | 7 | -31 | 68 | 1 | 75 | 0.9558 | 0.0224 | Y |
| CJDm | 16 | Cousens *et al*. 199721 | UK1970-96 | 3 | -15 | 64 | 0 | 65 | 0.7635 | 0.0101 | Y |
| CJDf | 17 | Cousens *et al*. 199721 | UK1970-96 | 3 | -15 | 64 | 0 | 65 | 0.9088 | 0.0009 | Y |

**Table S2. Number of studies per disease.** ‘Sources’ means number of articles or number of public databases with epidemiological data about the disease. ‘#’ means number of epidemiological studies in each source. Note that in the row of ‘Total sources’, the number of items is smaller than the sum of sources since a source can have data about more than one disease.

| **Dis.** | **Sources** | **#** |
| --- | --- | --- |
| AD | Andersen *et al*. 19993; Andreasen *et al*. 19994; Bermejo-Pareja *et al*. 200811; Chandra *et al*. 200118; Di *et al*. 200223; Edland *et al*. 200227; Ganguli *et al*. 200033; Gao *et al*. 199834; Hebert *et al*. 199542; Hendrie *et al*. 200144; Imfeld *et al*. 201348; Jorm & Jolley 199850; Kawas *et al*. 200052; Lobo *et al*. 201158; López-Pousa *et al*. 200461; Maestre *et al*. 201866; Matsui *et al*. 200968; Miech *et al*. 200269; Nilsson *et al*. 201473; Nitrini *et al*. 200474; Perez *et al*. 201077; Ravaglia *et al*. 200579; Ruitenberg *et al*. 200182; Tyas *et al*. 200692 | 30 |
| ADf | Bermejo-Pareja *et al*. 200811; Chandra *et al*. 200118; Di *et al*. 200223; Edland *et al*. 200227; Fratiglioni *et al*. 199732; Ganguli *et al*. 200033; Imfeld *et al*. 201348; Kawas *et al*. 200052; Kukull *et al*. 200254; Letenneur *et al*. 199956; Lobo *et al*. 201158; López-Pousa *et al*. 200461; Miech *et al*. 200269; Nitrini *et al*. 200474; Ravaglia *et al*. 200579; Rocca *et al*. 199880; Ruitenberg *et al*. 200182; Tyas *et al*. 200692 | 30 |
| ADm | Bermejo-Pareja *et al*. 200811; Chandra *et al*. 200118; Di *et al*. 200223; Edland *et al*. 200227; Fratiglioni *et al*. 199732; Ganguli *et al*. 200033; Imfeld *et al*. 201348; Kawas *et al*. 200052; Kukull *et al*. 200254; Letenneur *et al*. 199956; Lobo *et al*. 201158; López-Pousa *et al*. 200461; Miech *et al*. 200269; Nitrini *et al*. 200474; Ravaglia *et al*. 200579; Rocca *et al*. 199880; Ruitenberg *et al*. 200182; Tyas *et al*. 200692 | 30 |
| ALS | Aragones *et al*. 20165; Bettini *et al*. 201312; Caller *et al*. 201515; Cima *et al*. 200920; Golby *et al*. 201635; Henry *et al*. 201545; Joensen, 201249; Kab *et al*. 201751; Lareau-Trudel *et al*. 201355; Logroscino *et al*. 201059; Tesauro *et al*. 201788; Tobin *et al*. 201689; Wolf *et al*. 201498 | 20 |
| ALSf | Abhinav *et al*. 20071; Aragones *et al*. 20165; Benjaminsen *et al*. 201810; Bettini *et al*. 201312; Cima *et al*. 200920; Doi *et al*. 201424; Harper *et al*. 201541; Joensen, 201249; Kab *et al*. 201751; Logroscino *et al*. 201059; Marin *et al*. 201467; Rosenbohm *et al*. 201781; Tesauro *et al*. 201788; Traynor *et al*. 199990; Weil *et al*. 201696; Wolf *et al*. 201498; Zhou *et al*. 2018100 | 19 |
| ALSm | Abhinav *et al*. 20071; Aragones *et al*. 20165; Benjaminsen *et al*. 201810; Bettini *et al*. 201312; Cima *et al*. 200920; Doi *et al*. 201424; Harper *et al*. 201541; Joensen, 201249; Kab *et al*. 201751; Logroscino *et al*. 201059; Marin *et al*. 201467; Rosenbohm *et al*. 201781; Tesauro *et al*. 201788; Traynor *et al*. 199990; Weil *et al*. 201696; Wolf *et al*. 201498; Zhou *et al*. 2018100 | 19 |
| CJD | Begué *et al*. 20118; Elsaadany *et al*. 200528; Heinemann *et al*. 200743; Lu *et al*. 201060; Van *et al*. 201294 | 5 |
| CJDf | Cousens *et al*. 199721; Elsaadany *et al*. 200528; Gubbels *et al*. 201240; Heinemann *et al*. 200743; Lu *et al*. 201060; Nakamura *et al*. 199970 | 6 |
| CJDm | Cousens *et al*. 199721; Elsaadany *et al*. 200528; Gubbels *et al*. 201240; Heinemann *et al*. 200743; Lu *et al*. 201060; Nakamura *et al*. 199970 | 6 |
| DLB | Fleury *et al*. 201831; López-Pousa *et al*. 200362; Perez *et al*. 201077; Savica *et al*. 201384; Yang *et al*. 201899 | 5 |
| DLBf | Fleury *et al*. 201831; Savica *et al*. 201384 | 2 |
| DLBm | Fleury *et al*. 201831; Savica *et al*. 201384 | 2 |
| FTD | Andreasen *et al*. 19994; Knopman *et al*. 200453; López-Pousa *et al*. 200263; Nilsson *et al*. 201473 | 4 |
| HD | Chen & Lai 201019; Wexler *et al*. 201697 | 4 |
| HDf | Carrassi *et al*. 201717; Chen & Lai 201019 | 2 |
| HDm | Carrassi *et al*. 201717; Chen & Lai 201019 | 2 |
| MS | Barnett *et al*. 20037; Caniglia-Tenaglia *et al*. 201816; Gov. of Alberta, 201837; Nicoletti *et al*. 200172; Nicoletti *et al*. 201171; O’Connell *et al*. 201775; Palese *et al*. 201876; Sarasoja *et al*. 200483; Simonsen *et al*. 201785 | 28 |
| MSf | Barnett *et al*. 20037; Caniglia-Tenaglia *et al*. 201816; Gov. of Alberta, 201837; Hernán *et al*. 199946; Mackenzie *et al*. 201465; Nicoletti *et al*. 200172; Nicoletti *et al*. 201171; O’Connell *et al*. 201775; Palese *et al*. 201876; Sarasoja *et al*. 200483; Svenningsson *et al*. 199086 | 35 |
| MSm | Barnett *et al*. 20037; Caniglia-Tenaglia *et al*. 201816; Gov. of Alberta, 201837; Mackenzie *et al*. 201465; Nicoletti *et al*. 200172; Nicoletti *et al*. 201171; O’Connell *et al*. 201775; Palese *et al*. 201876; Sarasoja *et al*. 200483; Svenningsson *et al*. 199086 | 33 |
| PD | Alves *et al*. 20092; Baldereschi *et al*. 20006; Benito-Leon *et al*. 20049; Brewis *et al*. 196713; CPRD„ 201714; Das *et al*. 201022; Driver *et al*. 200925; Duncan *et al*. 201426; Evans *et al*. 201629; Fall *et al*. 199630; Fleury *et al*. 201831; Gordon *et al*. 201536; Gov. of Alberta, 201838; Granieri *et al*. 199139; Horsfall *et al*. 201347; Liu *et al*. 201657; MacDonald *et al*. 200064; Perez *et al*. 201077; Pupillo *et al*. 201678; Tan *et al*. 200787; Twelves *et al*. 200391; Van *et al*. 200393; Vines, 199995; de Lau *et al*. 2004101 | 58 |
| PDf | Alves *et al*. 20092; Baldereschi *et al*. 20006; CPRD„ 201714; Das *et al*. 201022; Duncan *et al*. 201426; Fleury *et al*. 201831; Gov. of Alberta, 201838; Pupillo *et al*. 201678; Tan *et al*. 200787; Van *et al*. 200393; de Lau *et al*. 2004101 | 28 |
| PDm | Alves *et al*. 20092; Baldereschi *et al*. 20006; CPRD„ 201714; Das *et al*. 201022; Duncan *et al*. 201426; Fleury *et al*. 201831; Gov. of Alberta, 201838; Pupillo *et al*. 201678; Tan *et al*. 200787; Van *et al*. 200393; de Lau *et al*. 2004101 | 29 |
| PDD | Savica *et al*. 201384 | 1 |
| PDDf | Savica *et al*. 201384 | 1 |
| PDDm | Savica *et al*. 201384 | 1 |
| PDM | Perez *et al*. 201077; de Lau *et al*. 2004101 | 2 |
| PDMf | de Lau *et al*. 2004101 | 1 |
| PDMm | de Lau *et al*. 2004101 | 1 |
| Total | 101 | 404 |

**Table S3. Parameters associated to the multistep model of the NDs based on stratified according to sex data.**

| **Nam** | **Steps** | **Risk** | **Age on set** | **Max incidence** | **Age max incidence** |
| --- | --- | --- | --- | --- | --- |
| AD | 11 | -43 | 79 | 2734 | 83 |
| ADf | 12 | -44 | 78 | 1987 | 80 |
| ADm | 10 | -36 | 77 | 1206 | 79 |
| ALS | 4 | -15 | 66 | 12 | 73 |
| ALSf | 4 | -15 | 66 | 8 | 71 |
| ALSm | 5 | -18 | 66 | 14 | 72 |
| CJD | 6 | -26 | 67 | 1 | 71 |
| CJDf | 5 | -24 | 67 | 0 | 72 |
| CJDm | 5 | -23 | 67 | 0 | 72 |
| DLB | 13 | -52 | 78 | 88 | 81 |
| DLBf | 11 | -43 | 75 | 23 | 75 |
| DLBm | 12 | -50 | 76 | 57 | 75 |
| HD | 1 | -4 | 51 | 1 | 50 |
| HDf | 1 | -6 | 50 | 0 | 47 |
| HDm | 1 | -6 | 55 | 1 | 47 |
| MS | 0 | 1 | 47 | 25 | 44 |
| MSf | 0 | 2 | 44 | 31 | 41 |
| MSm | 0 | 0 | 46 | 14 | 44 |
| PD | 5 | -18 | 72 | 187 | 77 |
| PDf | 5 | -17 | 71 | 129 | 77 |
| PDm | 5 | -18 | 71 | 251 | 77 |
| PDD | 10 | -42 | 80 | 47 | 85 |
| PDDf | 8 | -30 | 80 | 38 | 85 |
| PDDm | 10 | -40 | 81 | 68 | 85 |
| PDM | 6 | -18 | 80 | 911 | 86 |
| PDMf | 8 | -30 | 83 | 1020 | 90 |
| PDMm | 7 | -27 | 84 | 1210 | 90 |

**Table S4. Parameters associated to the multistep model of the NDs based on non-stratified according to sex data.**

| **Nam** | **Steps** | **Risk** | **Age onset** | **Max incidence** | **Age max incidence** |
| --- | --- | --- | --- | --- | --- |
| AD | 11 | -41 | 78 | 1976 | 81 |
| ALS | 4 | -16 | 66 | 11 | 72 |
| CJD | 6 | -25 | 67 | 0 | 72 |
| DLB | 12 | -50 | 77 | 67 | 78 |
| FTD | 5 | -18 | 68 | 28 | 74 |
| HD | 1 | -5 | 52 | 1 | 48 |
| MS | 0 | 1 | 46 | 24 | 43 |
| PD | 5 | -18 | 72 | 189 | 77 |
| PDD | 9 | -37 | 81 | 51 | 85 |
| PDM | 7 | -23 | 82 | 1013 | 88 |

## References

1. Abhinav, K. *et al*. Amyotrophic lateral sclerosis in South-East England: a population-based study. The South-East England register for amyotrophic lateral sclerosis (SEALS Registry). *Neuroepidemiology.* **29**(1-2), 44-8 (2007).
2. Alves, G. *et al*. Incidence of Parkinson’s disease in Norway: the Norwegian ParkWest study. *J Neurol Neurosurg Psychiatry.* **80**(8), 851-857 (2009).
3. Andersen, K. *et al*. Incidence of very mild to severe dementia and Alzheimer’s disease in Denmark: the Odense Study. *Neurology.* **52**(1), 85-90 (1999).
4. Andreasen, N. Blennow, K. Sjödin, C. Winblad, B. & Svärdsudd, K. Prevalence and Incidence of Clinically Diagnosed Memory Impairments in a Geographically Defined General Population in Sweden. *Neuroepidemiology.* **18**(3), 144-155 (1999).
5. Aragones, J.M. *et al*. Amyotrophic lateral sclerosis: A higher than expected incidence in people over 80 years of age. *Amyotroph Lateral Scler Frontotemporal Degener.* **17**(7-8), 522-7 (2016).
6. Baldereschi, M. *et al*. Parkinson’s disease and parkinsonism in a longitudinal study: two-fold higher incidence in men. ILSA Working Group. Italian longitudinal. *Neurology.* **55**(9), 1358–63 (2000).
7. Barnett, M.H. Williams, D.B. Day, S. Macaskill, P. & McLeod, J.G. Progressive increase in incidence and prevalence of multiple sclerosis in Newcastle, Australia: a 35-year study. *J Neurol Sci.* **213**(1-2), 1-6 (2003).
8. Begué, C. *et al*. Creutzfeldt-Jakob Disease Surveillance in Argentina, 1997–2008. *Neuroepidemiology.* **37**(3-4),193-202 (2011).
9. Benito-Leon, J. *et al*. Incidence of Parkinson disease and parkinsonism in three elderly populations of central Spain. *Neurology.* **62**(5), 734–41 (2004).
10. Benjaminsen, E. Alstadhaug, K.B. Gulsvik, M. Baloch, F.K. & Odeh, F. Amyotrophic lateral sclerosis in Nordland county, Norway, 2000-2015: prevalence, incidence, and clinical features. *Amyotroph Lateral Scler Frontotemporal Degener.* **19**(7-8), 522-7 (2018).
11. Bermejo-Pareja, F. Benito-León, J. Vega, S. Medrano, M.J. & Román GC; Neurological Disorders in Central Spain (NEDICES) Study Group. Incidence and subtypes of dementia in three elderly populations of central Spain. *J Neurol Sci.* **264**(1-2), 63-72 (2008).
12. Bettini, M. Vicens, J. Giunta, D.H. Rugiero, M. & Cristiano, E. Incidence and prevalence of amyotrophic lateral sclerosis in an HMO of Buenos Aires, Argentina. *Amyotroph Lateral Scler Frontotemporal Degener.* **14**(7-8),598-603 (2013).
13. Brewis, M. Poskanzer, D.C. Rolland, C. & Miller, H. Neurological disease in an English city. *Acta Neurol. Scand. Suppl.* **42**, 1–89 (1967).
14. CPRD. The prevalence and incidence of Parkinson’s in the UK. *Results from the Clinical Practice Research Datalink Reference Report* (2017).
15. Caller, T.A. Andrews, A. Field, N.C. Henegan, P.L. & Stommel, E.W. The Epidemiology of Amyotrophic Lateral Sclerosis in New Hampshire, USA, 2004-2007. *Neurodegener Dis.* **15**(4), 202-206 (2015).
16. Caniglia-Tenaglia, M. *et al*. Multiple sclerosis in the Republic of San Marino, Italian peninsula: an incidence and prevalence study from a high-risk area. *Neurol Sci.* **39**(7),1231-1236 (2018).
17. Carrassi, E. *et al*. Epidemiological study of Huntington’s Disease in the Province of Ferrara, Italy. *Neuroepidemiology.* **49**(1-2), 18-23 (2017).
18. Chandra, V. *et al*. Incidence of Alzheimer’s disease in a rural community in India: the Indo-US study. *Neurology.* **57**(6), 985-989 (2001).
19. Chen, Y.Y. & Lai, C.H. Nationwide population-based epidemiologic study of Huntington’s Disease in Taiwan. *Neuroepidemiology.* **35**(4), 250-254 (2010).
20. Cima, V. *et al*. Epidemiology of ALS in Padova district, Italy, from 1992 to 2005. *Eur J Neurol.* **16**(8), 920-924 (2009).
21. Cousens, S.N. *et al*. Sporadic Creutzfeldt-Jakob disease in the United Kingdom: analysis of epidemiological surveillance data for 1970-96. *BMJ.* **315**(7105), 389-395 (1997).
22. Das, S.K. *et al*. Epidemiology of Parkinson disease in the city of Kolkata, India. *Neurology.* **75**(15), 1362–1369 (2010).
23. Di Carlo, A. *et al*. Incidence of dementia, Alzheimer’s disease, and vascular dementia in Italy. The ILSA Study. *J Am Geriatr Soc.* **50**(1), 41-48 (2002).
24. Doi, Y. Atsuta, N. Sobue, G. Morita, M. & Nakano, I. Prevalence and incidence of amyotrophic lateral sclerosis in Japan. *J Epidemiol.* **24**(6), 494-499 (2014).
25. Driver, J.A. Logroscino, G. & Kurth, T. Incidence and remaining lifetime risk of Parkinson disease in advanced age. *Neurology.* 72,432-438 (2009).
26. Duncan, G.W. *et al*. The incidence of Parkinson’s disease in the North-East of England. *Age Ageing.* **43**(2), 257-263 (2014).
27. Edland, S.D. Rocca, W.A. Petersen, R.C. Cha, R.H. & Kokmen, E. Dementia and Alzheimer disease incidence rates do not vary by sex in Rochester, Minn. *Arch Neurol.* **59**(10),1589-1593 (2002).
28. Elsaadany, S. Semenciw, R. Ricketts, M. Mao, Y. & Giulivi, A. Epidemiological study of Creutzfeldt-Jakob disease death certificates in Canada, 1979-2001. *Neuroepidemiology.* **24**(1-2), 15-21 (2005).
29. Evans, J.R. *et al*. Comparative epidemiology of incident Parkinson’s disease in Cambridgeshire, UK. *J Neurol Neurosurg Psychiatry.* **87**(9), 1034-1036 (2016).
30. Fall, P.A. *et al*. Age-standardized incidence and prevalence of Parkinson’s disease in a Swedish community. *J. Clin. Epidemiol.* **49**(6), 637–41 (1996).
31. Fleury, V. Brindel, P. Nicastro, N. & Burkhard, P.R. Descriptive epidemiology of parkinsonism in the Canton of Geneva, Switzerland. *Parkinsonism Relative Disorders.* **54**,30-39 (2018).
32. Fratiglioni, L. *et al*. Very old women at highest risk of dementia and Alzheimer’s disease: incidence data from the Kungsholmen Project, Stockholm. *Neurology.* **48**(1),132-138 (1997).
33. Ganguli, M. Dodge, H.H. Chen, P. Belle, S. & DeKosky, S.T. Ten-year incidence of dementia in a rural elderly US community population: the MoVIES Project. *Neurology.* **54**(5),1109-1116 (2000).
34. Gao, S. Hendrie, H.C. Hall, K.S. & Hui, S. The relationships between age, sex, and the incidence of dementia and Alzheimer disease: a meta-analysis. *Arch Gen Psychiatry.* **55**(9),809-815 (1998).
35. Golby, R. *et al*. Five-year incidence of amyotrophic lateral sclerosis in British Columbia (2010-2015). *Can J Neurol Sci.* **43**(6), 791-795 (2016).
36. Gordon, P.H. *et al*. Incidence and prevalence of Parkinson’s disease among Navajo people living in the Navajo nation. *Mov Disord.* **30**(5), 714-720 (2015).
37. Gov. of Alberta-Canada. Multiple Sclerosis - Age-Sex Specific Incidence Rate. *www.ahw.gov.ab.ca/IHDA_Retrieval/* (2018).
38. Gov. of Alberta-Canada. Parkinson’s Disease - Age-Sex Specific Incidence Rate. *www.ahw.gov.ab.ca/IHDA_Retrieval/* (2018).
39. Granieri, E. *et al*. Parkinson’s disease in Ferrara, Italy, 1967 through 1987. *Arch. Neurol.* **48**(8), 854–57 (1991).
40. Gubbels, S. *et al*. Description and analysis of 12 years of surveillance for Creutzfeldt–Jakob disease in Denmark, 1997 to 2008. *Euro Surveill.* **17**(15) (2012).
41. Harper, C.J. Sorenson, E.J. & Mandrekar, J. Epidemiology of amyotrophic lateral sclerosis in Minnesota: a year-long population based study. *Amyotroph Lateral Scler Frontotemporal Degener.* **16**(7-8), 520-3 (2015).
42. Hebert, L.E. *et al*. Age-specific incidence of Alzheimer’s disease in a community population. *JAMA.* **273**(17), 1354-1359 (1995).
43. Heinemann, U. *et al*. Creutzfeldt-Jakob disease in Germany: a prospective 12-year surveillance. *Brain.* **130**(Pt 5), 1350-1359 (2007).
44. Hendrie, H.C. *et al*. Incidence of dementia and Alzheimer disease in 2 communities: Yoruba residing in Ibadan, Nigeria, and African Americans residing in Indianapolis, Indiana. *JAMA.* **285**(6), 739-747 (2001).
45. Henry, K.A. Fagliano, J. Jordan, H.M. Rechtman, L. & Kaye, W.E. Geographic Variation of Amyotrophic Lateral Sclerosis Incidence in New Jersey, 2009-2011. *Am J Epidemiol.* **182**(6), 512-519 (2015).
46. Hernán, M.A. Olek, M.J. & Ascherio, A. Geographic variation of MS incidence in two prospective studies of US women. *Neurology.* **53**(8), 1711-1718 (1999).
47. Horsfall, L. Petersen, I. & Walters, K. Time trends in incidence of Parkinson’s disease diagnosis in UK primary care. *J. Neurol.* **260**, 1351-1357 (2013).
48. Imfeld, P. Brauchli Pernus, Y.B. Jick, S.S. & Meier, C.R. Epidemiology, co-morbidities, and medication use of patients with Alzheimer’s disease or vascular dementia in the UK. *J Alzheimers Dis.* **35**(3), 565-573 (2013).
49. Joensen, P. Incidence of amyotrophic lateral sclerosis in the Faroe Islands. *Acta Neurol Scand.* **126**,62–66 (2012).
50. Jorm, A.F. & Jolley, D. The incidence of dementia: a meta-analysis. *Neurology.* **51**(3), 728-733 (1998).
51. Kab, S. Moisan, F. Preux, P.M. Marin, B. & Elbaz, A. Nationwide incidence of motor neuron disease using the French health insurance information system database. *Amyotroph Lateral Scler Frontotemporal Degener.* **18**(5-6), 426-33 (2017).
52. Kawas, C. Gray, S. Brookmeyer, R. Fozard, J. & Zonderman, A. Age-specific incidence rates of Alzheimer’s disease: the Baltimore Longitudinal Study of Aging. *Neurology.* **54**(11),2072-2077 (2000).
53. Knopman, D.S. Petersen, R.C. Edland, S.D. Cha, R.H. & Rocca, W.A. The incidence of frontotemporal lobar degeneration in Rochester, Minnesota, 1990 through 1994. *Neurology* **62**(3), 506-508 (2004).
54. Kukull, W.A. *et al*. Dementia and Alzheimer disease incidence: a prospective cohort study. *Arch Neurol.* **59**(11), 1737-1746 (2002).
55. Lareau-Trudel, E. *et al*. Epidemiological surveillance of amyotrophic lateral sclerosis in Saguenay region. *Can J Neurol Sci.* **40**(5),705-709 (2013).
56. Letenneur, L. *et al*. Are sex and educational level independent predictors of dementia and Alzheimer’s disease? Incidence data from the PAQUID project. *J Neurol Neurosurg Psychiatry.* **66**(2), 177-183 (1999).
57. Liu, W.M. *et al*. Time trends in the prevalence and incidence of Parkinson’s disease in Taiwan: A nationwide, population-based study. *J Formos Med Assoc.* **115**(7), 531-538 (2016).
58. Lobo, A. *et al*. Incidence and lifetime risk of dementia and Alzheimer’s disease in a Southern European population. *Acta Psychiatr Scand.* **124**(5), 372-383 (2011).
59. Logroscino, G. *et al*. Incidence of Amyotrophic Lateral Sclerosis in Europe. *J Neurol Neurosurg Psychiatry.* **81**(4), 385–39 (2010).
60. Lu, C.J. Sun, Y. & Chen, S.S. Incidence of Creutzfeldt-Jakob disease in Taiwan: a prospective 10-year surveillance. *Eur J Epidemiol.* **25**(5),341-347 (2010).
61. López-Pousa, S. Vilalta-Franch, J. Llinàs-Regla, J. Garre-Olmo, J. & Román, G.C. Incidence of dementia in a rural community in Spain: the Girona cohort study. *Neuroepidemiology.* **23**(4), 170-177 (2004).
62. López-Pousa, S. *et al*. Clinical incidence of dementia with Lewy bodies. *Rev Neurol.* **36**(8), 715-720. Spanish (2003).
63. López-Pousa, S. *et al*. [The clinical incidence of frontal dementia]. *Rev Neurol.* **34**(3), 216-222. Spanish (2002).
64. MacDonald, B.K. Cockerell, O.C. Sander, J.W. & Shorvon, S.D. The incidence and lifetime prevalence of neurological disorders in a prospective community-based study in the UK. *Brain.* **123**(Pt. 4), 665–76 (2000).
65. Mackenzie, I.S. Morant, S.V. Bloomfield, G.A. MacDonald, T.M. & O’Riordan, J. Incidence and prevalence of multiple sclerosis in the UK 1990-2010: a descriptive study in the General Practice Research Database. *J Neurol Neurosurg Psychiatry.* **85**(1), 76-84 (2014).
66. Maestre, G.E. *et al*. Incidence of dementia in elderly Latin Americans: Results of the Maracaibo Aging Study. *Alzheimers Dement.* **14**(2), 140-147 (2018).
67. Marin, B. *et al*. Population-based epidemiology of amyotrophic lateral sclerosis (ALS) in an ageing Europe–the French register of ALS in Limousin (FRALim register). *Eur J Neurol.* **21**(10), 1292-300, e78-9 (2014).
68. Matsui, Y. *et al*. Incidence and survival of dementia in a general population of Japanese elderly: the Hisayama study. *J Neurol Neurosurg Psychiatry.* **80**(4), 366-370 (2009).
69. Miech, R.A. *et al*. Incidence of AD may decline in the early 90s for men, later for women: The Cache County study. *Neurology.* **58**(2), 209-218 (2002).
70. Nakamura, Y. *et al*. Incidence rate of Creutzfeldt-Jakob disease in Japan. *Int J Epidemiol.* **28**(1), 130-134 (1999).
71. Nicoletti, A. *et al*. Increasing frequency of multiple sclerosis in Catania, Sicily: a 30-year survey. *Mult Scler.* **17**(3), 273-280 (2011).
72. Nicoletti, A. *et al*. Prevalence and incidence of multiple sclerosis in Catania, Sicily. *Neurology.* **56**(1), 62-66 (2001).
73. Nilsson, C. Landqvist Waldö, M. Nilsson, K. Santillo, A. & Vestberg, S. Age-related incidence and family history in frontotemporal dementia: data from the Swedish Dementia Registry. *PLoS One.* **9**(4), e94901 (2014).
74. Nitrini, R. *et al*. Incidence of dementia in a community-dwelling Brazilian population. *Alzheimer Dis Assoc Disord.* **18**(4), 241-246 (2004).
75. O’Connell, K. Tubridy, N. Hutchinson, M. & McGuigan, C. Incidence of multiple sclerosis in the Republic of Ireland: A prospective population-based study. *Mult Scler Relat Disord.* **13**,75-80 (2017).
76. Palese, F. *et al*. Epidemiology of amyotrophic lateral sclerosis in Friuli-Venezia Giulia, North-Eastern Italy, 2002-2014: a retrospective population-based study. *Amyotroph Lateral Scler Frontotemporal Degener.* **15**, 1-10 (2018).
77. Perez, F. Helmer, C. Dartigues, J.F. Auriacombe, S. & Tison, F. A 15-year population-based cohort study of the incidence of Parkinson’s disease and dementia with Lewy bodies in an elderly French cohort. *J Neurol Neurosurg Psychiatry.* **81**(7),742-746 (2010).
78. Pupillo, E. *et al*. Epidemiology of Parkinson’s Disease: A Population-Based Study in Primary Care in Italy. *Neuroepidemiology.* **47**(1), 38-45 (2016).
79. Ravaglia, G. *et al*. Incidence and etiology of dementia in a large elderly Italian population. *Neurology.* **64**(9), 1525-1530 (2005).
80. Rocca, W.A. Cha, R.H. Waring, S.C. & Kokmen, E. Incidence of dementia and Alzheimer’s disease: a reanalysis of data from Rochester, Minnesota, 1975-1984. *Am J Epidemiol.* **148**(1), 51-62 (1998).
81. Rosenbohm, A. *et al*. Epidemiology of amyotrophic lateral sclerosis in Southern Germany. *J Neurol.* **264**(4), 749-757 (2017).
82. Ruitenberg, A. Ott, A. van Swieten, J.C. Hofman, A. & Breteler, M.M. Incidence of dementia: does gender make a difference?. *Neurobiol Aging.* **22**(4), 575-580 (2001).
83. Sarasoja, T. Wikström, J. Paltamaa, J. Hakama, M. & Sumelahti, M.L. Occurrence of multiple sclerosis in central Finland: a regional and temporal comparison during 30 years. *Acta Neurol Scand.* **110**(5), 331-336 (2004).
84. Savica, R. *et al*. Incidence of dementia with Lewy bodies and Parkinson disease dementia. *JAMA Neurol.* **70**(11), 1396-1402 (2013).
85. Simonsen, C.S. Edland, A. Berg-Hansen, P. & Celius, E.G. High prevalence and increasing incidence of multiple sclerosis in the Norwegian county of Buskerud. *Acta Neurol Scand.* **135**(4), 412-418 (2017).
86. Svenningsson, A. Runmarker, B. Lycke, J. & Andersen, 0. Incidence of MS during two fifteen-year periods in the Gothenburg region of Sweden. *Acta Neurol Scand* **82**, 161-168 (1990).
87. Tan, L.C.S. Venketasubramanian, N. Jamora, R.D.G. & Heng, D. Incidence of Parkinson’s disease in Singapore. Parkinsonism and related disorders. *Parkinsonism Relat Disord.* **13**, 40-43 (2007).
88. Tesauro, M. *et al*. Incidence of amyotrophic lateral sclerosis in the province of Novara, Italy, and possible role of environmental pollution. *Amyotroph Lateral Scler Frontotemporal Degener.* **18**(3-4), 284-90 (2017).
89. Tobin, K. *et al*. Age-period-cohort analysis of trends in amyotrophic lateral sclerosis incidence. *J Neurol.* **263**(10), 1919-1926 (2016).
90. Traynor, B.J. *et al*. Incidence and prevalence of ALS in Ireland, 1995-1997: a population-based study. *Neurology.* **52**(3), 504-509 (1999).
91. Twelves, D. Perkins, K.S. & Counsell, C. Systematic review of incidence studies in Parkinson’s Disease. *Mov Disord.* **18**(1), 19-31 (2003).
92. Tyas, S.L. Tate, R.B. Wooldrage, K. Manfreda, J. & Strain, L.A. Estimating the incidence of dementia: the impact of adjusting for subject attrition using health care utilization data. *Ann Epidemiol.* **16**(6), 477-484 (2006).
93. Van Den Eeden, S.K. *et al*. Incidence of Parkinson’s disease: variation by age, gender, and race/ethnicity. *Am J Epidemiol.* **157**(11), 1015-1022 (2003).
94. Van Everbroeck, B. *et al*. Increased incidence of sporadic Creutzfeldt-Jakob disease in the age groups between 70 and 90 years in Belgium. *Eur J Epidemiol.* **21**(6),443-447 (2012).
95. Vines, J.J. Incidence of idiopathic and secondary Parkinson disease in Navarre. Population-based case registry. *Neurologica* **14**(1), 16–22 (1999).
96. Weil, C. Zach, N. Rishoni, S. Shalev, V. & Chodick, G. Epidemiology of amyotrophic lateral sclerosis: A population-based study in Israel. *Neuroepidemiology.* **47**(2), 76-81 (2016).
97. Wexler, N.S. *et al*. Incidence of adult Huntington’s disease in the UK: a UK-based primary care study and a systematic review. *BMJ Open.* **6**(2), e009070 (2016).
98. Wolf, J. *et al*. Incidence of amyotrophic lateral sclerosis in Rhineland-Palatinate, Germany. *Amyotroph Lateral Scler Frontotemporal Degener.* **15**(3-4), 269-74 (2014).
99. Yang, S.K. Chen, W. Su, C.H. & Liu, C.H. Incidence and comorbidity of Dementia with Lewy Bodies: A Population-based cohort study. *Behav Neurol.* **2018**, 7631951 (2018).
100. Zhou, S. *et al*. Using the capture-recapture method to estimate the incidence of amyotrophic lateral sclerosis in Beijing, China. *Neuroepidemiology.* **50**(1-2), 29-34 (2018).
101. de Lau, L.M. *et al*. Incidence of parkinsonism and Parkinson disease in a general population: The Rotterdam Study. *Neurology.* **63**(7), 1240-1244 (2004).
